# Amphotericin B Nano-Assemblies Circumvent Intrinsic Toxicity and Ensure Superior Protection in Experimental Visceral Leishmaniasis with Feeble Toxic Manifestation

**DOI:** 10.3390/vaccines11010100

**Published:** 2023-01-01

**Authors:** Fauzia Jamal, Ishrat Altaf, Ghufran Ahmed, Sheikh Asad, Hira Ahmad, Qamar Zia, Asim Azhar, Saba Farheen, Taj Shafi, Shabana Karim, Swaleha Zubair, Mohammad Owais

**Affiliations:** 1 Interdisciplinary Biotechnology Unit, Aligarh Muslim University, Aligarh 202002, India; 2 Department of Microbiology, Rajendra Memorial Research Institute of Medical Sciences, Patna 800007, India; 3 Department of Zoology, Aligarh Muslim University, Aligarh 202002, India; 4 Neat Meatt Biotech Private Limited, Bio-NEST-UDSC, University of Delhi (South Campus), New Delhi 110021, India; 5 Department of Molecular Biology, Rajendra Memorial Research Institute of Medical Sciences, Patna 800007, India; 6 Department of Botany, Anugrah Narayan College, Patliputra University, Patna 800013, India; 7 Department of Computer Science, Aligarh Muslim University, Aligarh 202002, India

**Keywords:** nano-assembled anti-leishmanial drug, green synthesis, Th_1_ polarization, nanoparticle, nano assembly, drug delivery

## Abstract

In spite of its high effectiveness in the treatment of both leishmaniasis as well as a range of fungal infections, the free form of the polyene antibiotic amphotericin B (AmB) does not entertain the status of the most preferred drug of choice in clinical settings. The high intrinsic toxicity of the principal drug could be considered the main impedance in the frequent medicinal use of this otherwise very effective antimicrobial agent. Taking into consideration this fact, the pharma industry has introduced many novel dosage forms of AmB to alleviate its toxicity issues. However, the limited production, high cost, requirement for a strict cold chain, and need for parenteral administration are some of the limitations that explicitly compel professionals to look for the development of an alternate dosage form of this important drug. Considering the fact that the nano-size dimensions of drug formulation play an important role in increasing the efficacy of the core drug, we employed a green method for the development of nano-assemblies of AmB (AmB-NA). The as-synthesized AmB-NA manifests desirable pharmacokinetics in the treated animals. The possible mechanistic insight suggested that as-synthesized AmB-NA induces necrosis-mediated cell death and severe mitochondrial dysfunction in *L. donovani* promastigotes by triggering depolarization of mitochondrial membrane potential. In vivo studies demonstrate a noticeable decline in parasite burden in the spleen, liver, and bone marrow of the experimental BALB/c mice host. In addition to successfully suppressing the *Leishmania donovani*, the as-formed AmB-NA formulation also modulates the host immune system with predominant Th1 polarization, a key immune defender that facilitates the killing of the intracellular parasite.

## 1. Introduction

Visceral leishmaniasis (VL) is highly endemic in South Asian countries, accounting for around ~60% of the total global burden, with a predominance in rural areas [1,2]. Countries such as India, Nepal, and Bangladesh are hotspots for the disease, with sporadic cases being reported in Bhutan and Sri Lanka [3]. VL has been considered one of the top most neglected parasitic diseases that poses a great health threat [4,5]. For example, the incidences of HIV in VL patients, and vice versa, are generally rampant, refractory, and difficult to treat, thereby calling for the specific attention of the medical fraternity to find a cure for this important disease [4,5].

The effective management of leishmaniasis relies on strategies that encompass both vector control as well as accessible chemotherapeutic options to effectively cure the disease [6]. Since therapy, with obsolete therapeutic drugs, is often marred with issues such as severe side effects, parasitic resistance, and enhanced post-kala-azar dermal leishmaniasis (PKDL) occurrence [3,7,8,9], repurposing of the drugs, such as AmB, could possibly result in the better clinical management of the disease [10].

Amphotericin-B, developed initially as an antifungal agent, manifests a great deal of toxicity in patients [11,12,13], whereas its lipid-based formulations (*cf*., AmBisome) have been considered as a first-line VL treatment option in endemic regions [5]. The liposomised AmB has been reported to cure the vast majority of VL cases as well as systemic fungal infections with significant decrement in intrinsic toxic manifestations [14]. However, global access to liposomised AmB is limited because of the manufacturing monopoly. For example, the supply of AmBisome has been channelized from a single pharmaceutical firm, GILEAD, the company responsible for manufacturing the innovative liposomal formulation. The limited production and supply (as witnessed during the current mucormycosis (black fungus) outbreak amidst the second wave of COVID-19 pandemic in India), high cost, the requirement for a strict cold chain, as well as need for parenteral administration hampered its worldwide availability [15,16,17]. Moreover, VL relapses with L-AmB treatment failure have been considered a matter of great concern worldwide [16,18,19].

Considering the limitations associated with lipid-based nano-formulations, there is a trend of developing some alternate nanosized drug delivery vehicles of AmB and a range of other drugs. In this regard, non-carrier-based nanosized drug assemblies have been found to manifest more discrete advantages in action as they do not manifest limitations or drawbacks associated with excipients [20]. Interestingly, the excipient-free nano-formulation ensures sustained and controlled release of the core drug. The minimal plasma level of the drug does not display drug allied toxic manifestations in the host compared to natural/generic forms of the drug [21]. As AmB toxicity depends on its aggregation state, it is conceivable that by regulating the degree of AmB aggregation, its cytotoxicity constraints can be alleviated [22,23]. 

Green synthesis of nanoparticles [24,25] proposes an attractive approach to improve the pharmacokinetic/pharmacodynamic profile of medicinally effective therapeutic agents. The particulate nature with specific size dimensions of the nanoparticle-based formulations effectively modulates intrinsic pharmacological properties of the core drug molecules [26,27,28]. Despite profound ramifications, limited reports are available on biologically influenced preparation of organic molecules-based nanoparticles. The plants (*Aloe vera*, *Azadirachta indica, Mangifera indica*), as well as microbes (*Candida albicans*, *Staphylococcus aureus*), offer an enormous tool with ever-increasing and innovative ways for the fabrication of nano-structured entities [29,30,31,32,33]. Noticeably, recent studies advocated the potent anti-inflammatory, anti-carcinogenic, anti-trypanosoma, anti-plasmodium, and anti-leishmanial activity of various parts of the *Mangifera indica* plant [34,35]. Previously held investigational verdict strongly advocated that plant extract fabricated nanoparticles are substantially active in arresting the development of promastigote and/or amastigote forms of *Leishmania donovani* [36,37] and *Leishmania tropica* [38,39,40]. The presence of bioactive substances such as polyphenols, vitamin C, carotenoids, anthocyanin, and flavonoids [41] in the employed plant extracts could be considered as the potent reason underneath that not only facilitate particle fabrication, but manifests antiparasiticidal activity as well. 

Furthermore, the anti-inflammatory, antioxidant, and immunomodulatory action of the compounds abundant in mango fruit juice further authenticated its antimicrobial potential [42,43,44,45]. Hence, owing to its to eco-friendly character, affordability, reduced hazardous nature, and presence of phytoconstituents that accentuate encapsulation, consistency and stability to the as-synthesized nanoparticles, plant extract-based green synthesis of nano-delivery systems offers an attractive method of nano-formulation fabrication [39]. Unfortunately, the vaccine against leishmaniasis is still not a reality; efforts are needed towards the improvement of existing drug regimens and the discovery of newer therapeutics to aid the fight against leishmaniasis.

In our previous studies, we explored the green synthesis of organic molecule-based nanoparticles. The as-synthesized nanoparticles were successful in increasing the therapeutic efficacy of the core drug in the treatment of cancer as well as a range of infectious diseases. The *Mangifera indica* plant has been reported to possess anti-inflammatory and immunomodulatory potential in general. Considering this fact, we harnessed mango fruit (*Mangifera indica*) pulp-mediated green synthesis of AmB-NA and explored its anti-parasitic potential against experimental VL. The as-synthesized AmB-NA possessed desirable physicochemical properties in terms of their size and surface features and elicited minimal toxic manifestations against living cells. 

## 2. Material and Methods

### 2.1. Chemicals and Reagents

All chemicals and reagents were procured from Sigma-Aldrich (St. Louis, MO, USA) unless otherwise mentioned. Other reagents were the AV apoptosis detection kit (BD Biosciences, San Jose, CA, USA, Cat. No. 556547, 556463), ELISA (BD Opt EIA^TM^) kits, DNA purification kit (Qiagen, Hilden, Germany, Cat. No. 51306), CytoTox test kit (Promega), 2′,7′-dichlorodihydrofluorescein diacetate, 0.14 M K_3_PO_4_, 0.14 M Na_3_PO_4_, 3 mM KCl, and 0.14 M NaCl, etc.

### 2.2. Animal Model and Experimental Design

The in vivo experiments were performed as per the procedures laid out for the Care and Use of Laboratory Animals (Institute for Laboratory Research, 2011) under the approved protocol by the institutional animal ethics committee (IAEC) of the Indian Council of Medical Research, Rajendra Memorial Research Institute of Medical Science (ICMR-RMRIMS), India (RMRI/AH/IAEC/05/2019-20). The animals were kept in polycarbonate cages (standard size) in a group of at least six mice in the animal facility of ICMR-RMRIMS, Patna, India. The temperature (25 ± 2 deg C), light hours (12 h dark 12 h light cycle), and humidity (55 ± 10%) were properly maintained. As per instructions, food and water were provided, and the health of the animals was regularly checked.

We upheld humane endpoints for the mice that survived after the termination of the study. Intraperitoneal administration of an anesthetic cocktail of ketamine (5 mg/kg) and xylazine (4 mg/kg) in the experimental animals (mice) was conducted. Mice were euthanized by cervical dislocation. Experiments involving the injection, bleeding, and sacrifice of animals rigorously followed the guidelines approved by the IAEC as per the recommendations of CPCSEA, Govt. of India. All efforts were made to lessen the pain and suffering of the experimental animals while performing the experiment.

### 2.3. Preparation of Mangifera Indica Fruit Extract

Briefly, the *Mangifera indica* fruit was washed thoroughly, and the fruit pulp (30 g fruit pulp) was cut into small pieces followed by homogenization using an electric blender. The macerated fruit pulp was boiled with 100 mL of MilliQ water. Thereafter, the fruit pulp was allowed to cool at room temperature (RT) and was filtered through the Whatman filter; finally, the filtrate was stored at −20 °C until further use. 

### 2.4. Biomimetic Synthesis of Nano-Assembled Amphotericin B (AmB-NA)

The methodology for the preparation of *Mangifera indica* extract, synthesis of AmB-NA, and their physiochemical characterization was done according to the protocols mentioned elsewhere [21]. Briefly, AmB-NA was prepared and standardized by taking 5 mL of *Mangifera indica* pulp extract (30% *w*/*v* stock solution in MilliQ water) and mixing it with 5 mL Amphotericin B (AmB) (1 mM in dimethyl sulfoxide [DMSO]) to obtain a final volume of 10 mL in MilliQ water. The mixture was stirred at RT for a specified time (24 h). At different time intervals, aliquots of said cocktail were taken for spectrophotometric assessment. In another set of experiments, variations in the AmB super-aggregates formation were measured using increasing concentrations of *Mangifera indica* pulp extract (1 mL, 3 mL, and 5 mL). Subsequently, the suspension was centrifuged at 20,000× *g* for 20 min at 4 °C to pellet down AmB-NAs. The obtained pellet was washed with PBS (three times) and finally resuspended in PBS (5 mL). The suspension was subjected to dialysis for about 24 h against 1 L PBS (twice) to remove monomeric AmB. Finally, the purified AmB-NA was freeze-dried (without a cryoprotectant) to yield a yellow fluffy product. The as-synthesized AmB-NA was characterized and analyzed for its anti-leishmanial activity. The detailed methodology for the physico-chemical characterization of the as-synthesized AmB-NA has been discussed in Supplementary Materials S1.

### 2.5. In Vitro Release Kinetics

The release of monomeric AmB from the as-formed AmB-NAs was assessed by dialysis in PBS buffer as per the protocol standardized in our lab (21). A 10 mL stock of AmB-NA (1 mg/2 mL) was prepared in 20 mM PBS. The AmB-NA was put into a dialysis bag (molecular weight cut off 12–14 kDa) and dialysis was done against 50 mL of 20 mM PBS with 5% DMSO on a magnetic stirrer maintained at 37 °C. After each 25-h time period, 2 ml volume of PBS was withdrawn and released free form of AmB was assessed by UV-VIS spectrophotometer at a wavelength of 408 nm. The exact amount of PBS was put into the solution that had been taken out. The pH was maintained at 7.4 during the entire dialysis process. The amount of drug released (in percentage) was calculated and plotted against time (in hours). 

The loading capacity was calculated by centrifugation of the as-formed nanoassembly for 30 min at 15,000× *g* at 25 °C. The sample subjected to centrifugation contained 1 mL of the nanoassembly mixed with 9 mL of DMSO. The loading capacity of the AmB nanoassembly was calculated from the formula:

(1) $$\mathrm{Loading}\;\mathrm{capacity}=\frac{(\mathrm{Weight}\;\mathrm{of}\;\mathrm{drug}\;\mathrm{in}\;\mathrm{nanoassembly}-\mathrm{Weight}\;\mathrm{of}\;\mathrm{drug}\;\mathrm{in}\;\mathrm{supernatant})\times 100}{\mathrm{Weight}\;\mathrm{of}\;\mathrm{drug}\;\mathrm{nanoassembly}}$$

### 2.6. Toxicity Assessment of the AmB-NA Formulation

A detailed procedure for the toxicity assessment was done following the experimental procedure stated previously [21,46]. 

#### 2.6.1. Haemolytic Effect of AmB-NA

The haemolytic effect of as-synthesized AmB-NA on erythrocytes (RBCs) was monitored as described earlier with minor modifications [26]. For this, the BALB/c mice (*n* = 3) were sacrificed by carbon dioxide (CO_2_) asphyxiation, and cardiac blood was harvested and processed for RBC retrieval by centrifugation at 500× *g* for 10 min at 4 °C. Isolated RBCs were adjusted (2 × 10^8^ cells/mL) using a Neubauer counting chamber. The RBCs were seeded in microtiter plates (100 µL/well) with increasing concentrations of AmB-NA at 37 °C for 24 h. In 50 μL of DMSO, the free form of AmB was dissolved and diluted to 1 mL with PBS (final 5% DMSO). For 100% cell lysis, triton X-100 (non-ionic surfactant) at a concentration of 0.1% was used as a positive control. After a specified time interval, the reaction mixture was centrifuged at 1200× *g*, the supernatant was collected, and the absorbance was recorded at 576 nm for released hemoglobin. The absorbance was used to derive percent hemolysis as a function of nanoparticle concentration. The result was expressed graphically as a percentage of 100% cell lysis and was determined by the following equation:

(2) $$\mathrm{Percent}\;\mathrm{hemolysis}=\frac{({\mathrm{Abs}}_{T}-{\mathrm{Abs}}_{C})\;\times \;100}{{\mathrm{Abs}}_{100\%}-{\mathrm{Abs}}_{C}}$$

where Abs_T_ is the absorbance from samples incubated with the drugs, Abs_C_ is the absorbance from the control (PBS), and Abs_100%_is absorbance in the presence of 0.1% Triton X-100. Each data point represents mean ± SD (triplicate determination).

#### 2.6.2. MTT Assay

The cytotoxic effect of the as-synthesized AmB-NA formulation was also evaluated against mouse peritoneal exudate cells (PECs) as previously reported with minor modifications [42]. For this, the BALB/c mice were humanely sacrificed, and PECs were isolated from peritoneal lavage in a sterile condition. The PECs were washed twice with sterile Hanks balanced salt solution (HBSS) by centrifugation at 500× *g* for 15 min at 4 °C and suspended in complete Roswell Park Memorial Institute (RPMI)-1640 medium. The cell density was adjusted (1 × 10^6^/mL) and cultured (200 µL/well) in a Lab-Tek^R^ chamber slide (Nunc^TM^) in a CO_2_ incubator (for 48 h at 37 °C). Next, the PECs were exposed to increasing concentrations of AmB-NA and incubated in a CO_2_ incubator for 24 h at 37 °C. Post incubation, the effect of AmB-NA on PECs was analyzed, employing microscopic analysis and MTT assay (3-(4,5-dimethylthiazol-2-yl)-2,5-diphenyltetrazolium bromide). The optical density of the colored complex was measured at 570 nm using a spectrophotometer (Bio-Rad, Hercules, CA, USA), plotted against nanoparticle concentration. 

The percentage of living cells was calculated using the following formula:

(3) $$\mathrm{Percent}\;\mathrm{cell}\;\mathrm{viability}=\frac{(\mathrm{Sample}\;\mathrm{absorbance}-\mathrm{blank}\;\mathrm{absorbance})\;\times \;100}{\mathrm{Control}\;\mathrm{absorbance}-\mathrm{blank}\;\mathrm{absorbance}}$$

where blank absorbance is the medium alone, and control absorbance are the cells with medium alone. All assays were performed in triplicate, and the results are expressed as the mean.

### 2.7. Leishmania Donovani Culture

For in vitro and in vivo investigations, a reference strain of *Leishmania donovani* (MHOM/IN/83/AG83) was retrieved from the ICMR-RMRIMS cryobank. Parasites were raised in RPMI-1640 artificial media supplemented with 10% heat-inactivated foetal bovine serum (FBS), penicillin (Sigma-Aldrich) (50 μg/mL, gentamicin (25 μg/mL), and streptomycin (50 μg/mL) and incubated at 25 °C to facilitate biological oxygen demand (BOD). Ficoll gradient was used to harvest metacyclic promastigotes. 

### 2.8. Parasiticidal Potential of As-Synthesized AmB-NA against L. donovani

The parasiticidal activity of the as-synthesized AmB-NA against intracellular amastigotes and promastigotes was evaluated as described earlier with slight modifications, as standardized in our laboratory [46]. Giemsa staining was used for the evaluation of amastigote activity. PECs-derived macrophages were challenged in the metacyclic stage of *L. donovani* (Ld) promastigotes at a macrophage to parasite ratio of 1:10 followed by incubation in a CO_2_ incubator for 48 h for their conversion to amastigotes. Non-internalized parasites were removed by gentle washing with an RPMI-1640 medium. Subsequently, infected macrophages were treated with increasing concentrations of AmB-NA (0.5–1.0 μM). The treated cells were further incubated for another 48 h with a complete RPMI-1640 medium. The untreated cells served as a negative control, and pure AmB drug-treated wells were considered as positive control. Next, Ld promastigotes (1 × 10^6^ cells/mL) were incubated with AmB-NA, AmBisome^®^, and Fungizone^®^ at various concentrations (0.05–1.0 µM) for 48 h, and cell viability was measured by microscopy and an MTT assay using standard procedures. The MTT experiment was performed by adding 10% MTT reagent to the culture medium followed by 4 h incubation. A corresponding amount of solubilizing agent was applied. The complete culture was transferred to a 96-well plate and mixed well, and the optical density was measured at 570 nm using an ELISA plate reader. After 48 h, the drug concentration that resulted in 50% parasite death was referred to as the IC_50_; proportionately, the drug concentration that resulted in 90% parasite killing was referred to as the IC_90_. Coherently, CC_50_ was also calculated which refers to the cytotoxic concentration of the formulations required to kill 50% of the viable macrophage. The selectivity index (SI = CC_50_/IC_50_) was calculated to prove the anti-leishmanial potential of the drug. SI is ideal to achieve a high therapeutic index that delivers the highest antileishmanial efficacy with the least amount of cell damage.

### 2.9. Effect of Drug on Promastigote Form of the Parasite

#### 2.9.1. Evaluation of Intracellular Reduced Thiol

Following treatment with various forms of AmB-NA formulations (at IC_50_ concentration.), the level of total intracellular reduced thiol was measured in deproteinized extracts of metacyclic *L*. *donovani* with or without the drug (administered at their IC_50_ concentration) following a method described elsewhere [46] with slight modification. Briefly, the propagated culture of *L. donovani* (2 × 10^6^ cells/mL) was harvested, washed twice in buffer (0.14 M K_3_PO_4_, 0.14 M Na_3_PO_4_, 3 mM KCl and 0.14 M NaCl), suspended in 0.6 mL of 25% TCA, and freeze-thawed for three cycles. Centrifugation was used to eliminate denatured proteins and cell debris. In total, 0.6 mM 5,5-dithio-bis 2-nitrobenzoic acid (DTNB, Ellman’s reagent) in 0.2 M Na_3_PO_4_ (pH 8.0) was used to determine the thiol content of the supernatant. The concentration of DTNB thiol derivatives was measured spectrophotometrically at 412 nm.

#### 2.9.2. DNA Fragmentation Assay

DNA fragmentation assay was performed according to a previously described protocol [47] as standardized in our lab. The parasites (1 × 10^6^ cells/mL) were incubated with as-synthesized AmB-NA as well as other control formulations such as AmBisome and free AmB for 6 h. We also included untreated and H_2_O_2_-treated (0.2 mM) parasites as negative and positive controls, respectively, in an experimental setup. For harvesting parasites, a fixed number of cells (1 × 10^7^) in the late log phase were centrifuged at 900× *g* for 20 min at 4 °C (Hermle, Germany). The pellets (10^8^ promastigotes) were washed with PBS thrice and centrifuged (900× *g*) at 4 °C for 20 min. The obtained pellet was used to isolate DNA using a commercial DNA purification kit (Qiagen, Germany, Cat. No. 51306). The isolated DNA aliquots (10 μL), representing various experimental groups, were electrophoresed on 0.8% agarose gel and visualized under UV illumination after ethidium bromide staining.

#### 2.9.3. Flow Cytometric Analysis of AmB-NA Treated Promastigotes Employing Annexin-V–FITC/Propidium Iodide

The potential of as-synthesized AmB-NA to induce parasite cell apoptosis was assessed based on Annexin V-FITC/propidium iodide (PI) binding of parasites employing AV apoptosis detection kit (BD Biosciences, San Jose, CA, USA, Cat. No. 556547, 556463). Briefly, promastigotes (1 × 10^6^ cells/mL) were administered with AmB-NA, AmBisome, and AMB, at their IC_50_ and IC_90_ for 4 h and 8 h, respectively. Untreated and H_2_O_2_-treated (0.2 mM) parasites were considered as negative and positive controls, respectively. Cells were washed, collected, and dissolved in PBS followed by mixing with a consecutive addition of 5 μL PI, 20 μL of binding buffer, and 5 μL of Annexin V-FITC. Following 15 min incubation, cells were assessed employing flow cytometry (FACS Calibur, Becton Dickinson, CA, USA) [44,48].

#### 2.9.4. Membrane Integrity Assay

Intracellular LDH production is linked to cell membrane deterioration and necrosis. The amount of LDH that leaked from the membrane of damaged cells were examined. In a nutshell, released lactate dehydrogenase was assessed from the cells with damaged membranes using the CytoTox test kit after untreated and treated promastigote parasites (1 × 10^6^ cells/mL) were incubated for 12 h (Promega). As a positive control, promastigote parasites treated with 1% TritonX-100 for 15 min revealed ample cell lysis.

#### 2.9.5. Analysis of Variation in Mitochondrial Membrane Potential (ΔΨ_m_)

The JC-1 (5,5′,6,6′-tetrachloro-1,1′,3,3′-tetraethylimidacarbocyanine iodide) dye (Invitrogen) [47] was used to evaluate the alteration in the ΔΨm of *L. donovani* membrane [49]. The JC-1 dye fluoresces differently within living and dead cells. It is kept inside living cells’ mitochondria in the form of J-aggregates, but in damaged cells, it is released from the mitochondria and dwells in the cytoplasm in a monomeric state (J-monomers). In general, the ratio of J aggregates (red) to J monomers (green) indicates the cell’s relative Ψm [50]. Briefly, to determine ΔΨ_m_ in Ld, the promastigotes (1 × 10^6 mL^/L) were incubated in the absence or presence of IC_50_ of AmB-NA for 6 h at 22 °C in a BOD incubator. AmB was also tested at the same concentration in parallel. Post-treatment, the cells were harvested, washed twice with PBS, and finally stained with JC-1 (10 μg, 20 min, 37 °C). Following labelling, the cells were washed twice with PBS, and fluorescence was measured using a FACS Caliber flow cytometer (excitation at 488 nm and emission at 530/585 nm for green and red channels, respectively). To ascertain the ΔΨm, we need to differentiate the red J-aggregates from J-monomers; the red (J-aggregates)/green (J-monomer) ratio (i.e., 585/530) was determined using the FlowJo software. 

### 2.10. Anti-Leishmanial Activity of AmB-NA in a Mouse VL Model

The efficacy of AmB-NA was assessed by monitoring the degree of *Leishmania* parasite clearance in a mouse VL model following the published protocol as modified in our lab [51]. For this, six- to eight-week-old healthy inbred BALB/c male mice (*n* = 42) were procured from the animal house of the institute (ICMR-RMRIMS, Patna) and divided into different groups consisting of six animals each (Group-I to VII). 

Group I, Control (Only PBS) (Negative control)

Group II, Infected (Positive Control)

Group III, *M. indica* pulp extract (MPE)

Group IV, Pure AmB 

Group V, AmBisome 

Group VI, AmB-NA (single dose) 

Group VII, AmB-NA (double dose)

All animals were intraperitoneally (IP) inoculated with *Leishmania* parasites (1 × 10^7^/mL), except Group-I, which served as the uninfected control. Confirmation of infection was evaluated in two arbitrarily selected mice six weeks post-infection. Subsequently, infected mice groups were treated IP with respective drug regimens AmB-NA at 10 mg/kg body weight (bw), AmBisome (10 mg/kg bw), and AmB (1 mg/kg bw) or PBS; AmBisome and AmB-NA was administered twice, at day 1 and day 16 (i.e., at a fifteen- day interval) in a volume of 100 µL, whereas for AmB, it was every alternate day for 15 days. At two weeks post-treatment, mice were humanely sacrificed, and the presence of *L. donovani* amastigotes was quantitatively analyzed by dabbing the liver, spleen, and bone marrow, followed by Giemsa staining. The parasitic burden was monitored microscopically by counting the number of amastigotes per 1000 fields. 

### 2.11. Soluble Leishmanial Antigen (SLA) Preparation

SLA antigen was obtained from stationary phase promastigotes [47]. Promastigotes were harvested in the third or fourth passage. Thereafter, they were washed four times in cold PBS (1X), and again suspended at 2 × 10^8^ cells/mL. For six cycles, the suspension was frozen at −80 °C (30 min) and thawed in a 37 °C water bath (15 min). After ten alternate freeze-thaw cycles, they were centrifuged (5250× *g*, 10 min at 4 °C) to harvest the supernatant comprising leishmanial antigens (SLA). SLA was kept at −80 °C, and prior to use the protein was quantified.

### 2.12. Assessment of the Delayed Type of Hypersensitivity (DTH) Response

DTH as a pointer of elicitation of cell-mediated immunity was noticed in all the experimental BALB/c mice 4 weeks post-treatment. Mice were inoculated with 50 μg of SLA (μg/μL) in the left hind footpad, and an equal volume of PBS (50 μL) was injected in the right hind footpad. The DTH response was assessed by measuring the differential footpad oedema (left-right) 48 h after antigen (SLA) treatment using Vernier callipers, as described previously [47,48,49,52,53]. 

### 2.13. Assessment of Serum Immunoglobulin (IgG) Isotype

To evaluate the IgG level, 4-week post-treatment blood from various experimental groups were collected to separate the sera. The concentrations of IgG_2a_ and IgG_1_ in the sera from uninfected, infected, and infected untreated mice were determined using an enzyme-linked immunosorbent assay (ELISA), as previously described [52]. In a nutshell, 96-well ELISA plates (Nunc, Rochester, NY, USA) were coated with 100 µL of SLA (5 µg/mL) in a bicarbonate buffer (pH 9.6) and incubated overnight at 4 °C. Next, the plates were blocked with 5% skimmed milk containing 1 × PBS (blocking buffer) for 1 h. Before adding the primary antibody (mice sera) at a 1:1000-fold dilution, the plates were rinsed thrice with 0.1% PBS-T, followed by washing. Afterwards, HRP conjugated anti-mouse IgG_1_ and IgG_2a_ secondary antibodies were added at a dilution of 1: 5000. Lastly, 3, 3′, 5, 5′-Tetramethylbenzidine (TMB) was added, and the reaction was stopped using 1N H_2_SO_4_ (50 µL/well). The absorbance was measured at 450 nm using an ELISA reader (Bio-Rad, Hercules, CA, USA).

### 2.14. AmB-NA Treatment Ensues in Th_1_/Th_2_ Cytokine Production

To assess the T helper type (Th_1_) stimulatory potential of AmB-NA in BALB/c mice, the extracellular cytokines were assessed by an enzyme-linked immunosorbent assay (ELISA) kit of mouse splenocytes as described earlier with minor changes [54]. Following sacrifice, spleens were aseptically harvested from normal, infected, and treated mice 4-week post-treatment. Spleens thus obtained were processed for procuring splenocytes single-cell suspension. The obtained splenocytes were cultured and incubated in a CO_2_ incubator at 37 °C. After 48 h of SLA stimulation, the cell-free culture supernatants from all experimental groups were collected and quantitively examined for cytokines (IL-10, IL-12, IFN-γ, TNF-α) using ELISA (BD OptEIA^TM^) kits as per the protocol supplied with the respective kit. Variation in various cytokines expression level was further authenticated by reverse transcription polymerase chain reaction (RT-PCR). Reverse transcription was executed using 1 μg of total RNA employing a cDNA synthesis kit (Roche, Carlsbad, CA, USA). The synthesized cDNA was amplified by PCR. The primer sequence for IFN-γ, IL-10, TNF-α, and GAPDH were as follows: Forward _IFNγ_: ATGAACGCTACACACTGCAT, Reverse _IFNγ_: AGTCTGAGGTAGAAAGAGAT, Forward _IL-10_: GGTGCCTATGTCTCAGCCTCTT Reverse _IL-10_: CCATAGAACTGATGAGAGGGAG, Forward _TNF-α_: ATGCCTGGCTCAGCACTGCT Reverse _TNF-α_: TAACCCTTAAAGTCCTGCAT, Forward _GAPDH_: TGCATCCTGCACCACCAACT, and Reverse _GAPDH_: TGGGATGACCTTGCCCACAG, respectively. Obtained product lengths were 230 bp, 220 bp, 230 bp, and 220 bp for IFN-γ, IL-10, TNF-α, and GAPDH, respectively (Supplementary Materials S2).

### 2.15. Determination of Reactive Nitrogen Species (RNS) and Reactive Oxygen Species (ROS) 

Evaluation of nitrites from amastigotes was done by using a Griess reagent and performing semi-quantitative PCR for genes specific for mice inducible nitric oxide synthase (iNOS) and GAPDH. Reverse transcription was executed using 1 μg of total RNA employing a cDNA synthesis kit (Roche, Carlsbad, CA, USA). The synthesized cDNA was amplified by PCR. The primers used for iNOS and GAPDH were as follows: Forward _iNOS_: AGGAGGAGAGAGATCCGATTTAG Reverse _iNOS_: TCAGACTTCCCTGTCTCAGTAG Forward _GAPDH_: TGCATCCTGCACCACCAACT, and Reverse _GAPDH_: TGGGATGACCTTGCCCACAG, respectively (Supplementary Materials S3). Obtained product lengths were 125 bp and 220 bp for IFN-γ, IL-10, TNF-α, and GAPDH, respectively, whereas, a ROS assay was performed by ELISA using H_2_DCFDA (2′,7′-dichlorodihydrofluorescein diacetate) (Supplementary Materials S4). 

### 2.16. Statistical Analysis

The experimental data presented in the figures are represented as means ± SEM and analyzed through Graph pad prism version 6.0. The significance was determined by one-way or two-way analysis of variance (ANOVA) with Tukey’s post hoc multiple comparison tests. All experiments were done in triplicates, and *p* ≤ 0.05 was considered statistically significant.

## 3. Results

### 3.1. Spectroscopic Analysis of As-Synthesized AmB-NA Formulation

**Figure 1:**
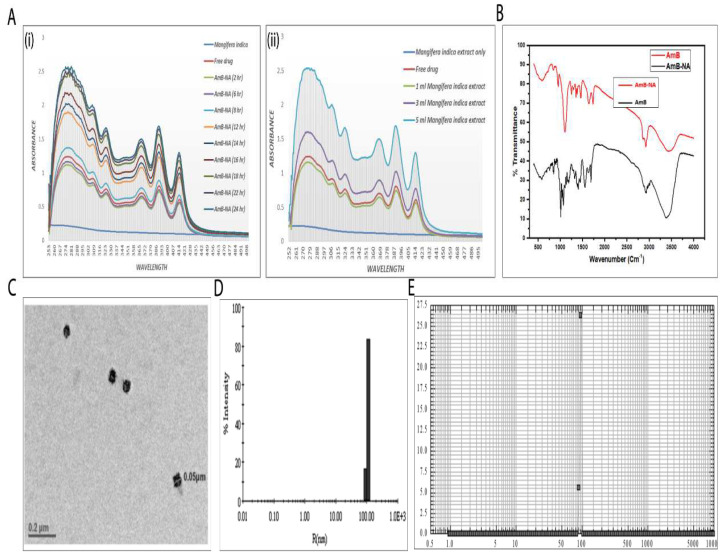
Physico-chemical characterization of the as-synthesized AmB-NA. The co-incubation of AmB with *Mangifera indica* fruit pulp resulted in the green synthesis of AmB-NAs. (**A**) Effect of various parameters influencing synthesis and physico-chemical properties of the as-synthesized AmB-NA (**i**) Time kinetics of AmB-NA synthesis as determined on the basis of the UV-VIS spectrophotometric analysis, (**ii**) Concentration-dependent kinetics of as-synthesized AmB-NA was monitored by co-incubation of 1 mM AmB with the increasing volume of the stock of *Mangifera indica* fruit pulp extract. (**B**) The IR-spectrum of the as-synthesized AmB-NAs showing characteristic functional groups of the parent AmB compound along with residues of fruit pulp in the as-synthesized AmB-NAs, (**i**) FTIR absorption spectra of as-synthesized AmB-NA formulation (**C**) Size distribution and morphology of the as-synthesized AmB-NA as revealed by representative TEM image of the as-fabricated AmB-NA synthesized by employing 5 mL of *Mangifera indica* pulp extract mixed with 5 mL of 1 mM AmB solution. (**D**) The average diameter of AmB-NA, determined by DLS measurements, was 97 ± 0.8 nm for AmB-NA. (**E**) Particle size distribution of AmB-NA assessed by photon correlation spectroscopy was estimated to be 101 ± 0.7 nm for AmB-NA.

The spectroscopic assessment suggested that as-synthesized AmB-NA retained the UV-absorption characteristics of the parent AmB compound and bears a resemblance to super-aggregates of AmB. A significant hypsochromic shift in the absorption spectrum of the as-synthesized AmB-NAs was monitored, with the highest peak around 280 nm. The time kinetic data of the synthesis revealed a similar characteristic spectrum at various time points where magnitude of the absorption peaks changed (first reduction until 6 h post initiation, followed by increment) with time. To avoid ambiguity, the free AmB (pure drug) absorption spectrum was noted immediately after its mixing with *Mangifera indica* pulp extract, considered as the zero time point observation. The interaction ensued quenching in the absorbance intensity of several characteristic absorption peaks of the AmB up to 6 h (Figure 1A(i)). A substantial elevation in intensity was detected after 8 h, which was greater than the intensity of free AmB at the zero time point. Altogether, slow nanoparticle formation was noticed and found to be accomplished in 24 h. After 18 h, no significant difference was noticed up to 24 h, and beyond that, it did not result in any further upsurge in optical density. To remove the mango extract trace, the AmB-NA thus formed were pelleted and washed carefully. Likewise, the consequences of increasing *Mangifera indica* pulp extract concentrations on nanoparticle super aggregation formation were monitored. At a fixed time (24 h), insignificant variation was observed in a reaction mixture containing AmB and mango pulp in the ratio of 1:5, whereas a significant uptick was noted with a 3:5 ratio (Figure 1A(ii)).

Further increments in mango pulp extract (5 mL) resulted in an abrupt upsurge in peak intensity. A hypochromic shift in the absorption spectrum of as-synthesized AmB-NAs (super aggregates) was detected upon incubation by raising the mango pulp extract volume. This proves that augmentation in the concentration of *Mangifera indica* pulp extract is actively involved in drug nano-assembly.

### 3.2. Physico-Chemical Characterization of AmB-NA Formulation

The IR spectra of AmB-NA (super aggregates) showed characteristic peaks of the parent drug (Figure 1B). The spectral region between 1500 and 1800 cm^−1^ represents the stretching vibrations of the C=O, —COO^−^, —NH_3_^+^, and C=C functional groups. There is an increase of the bandwidth in the range 3300–3500 cm^−1^, characteristic for hydrogen-bonded AmB molecules (—OH••••HO—). The formation of molecular pores is related to the influence of the hydroxyl groups, where corresponding bands appear in the region of deformational vibrations of the C—O bond. The bands in the range of 1330–1040 cm^−1^ are associated with stretching vibrations (deformation of C—O and C—O—C bonds) as well as C—H (deformation of out-of-plane vibrations) in the AmB chromophores characteristic of all-trans polyenes [55], which further indicates their participation in the nano-assembly formation. A sharp intense band centered at 1604, and 1657 cm^−1^ is clearly visible [52,56]. This is a direct indication of the asymmetric stretch of the –COO^−^ group in AmB [57].

The typical TEM image of AmB-NA showed the shape of the particles to be nearly spherical with an uneven surface morphology and narrow size distribution, with a size of ~50 nm (Figure 1C). The mean size of the nanoparticles was determined to be 97 ± 0.8 nm as measured by dynamic light scattering (DLS) (Figure 1D). This was in agreement with the average size determined by the nanosizer (average diameter 101 ± 0.7 nm, Figure 1E). Furthermore, the zeta potential of the nanoparticles AmB-NA was found to be −34.2 ± 0.3 mV (Table 1). 

### 3.3. In Vitro Drug Release Kinetics

**Figure 2:**
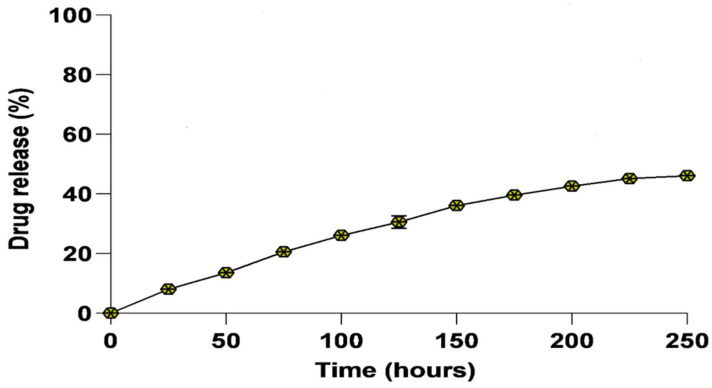
Release kinetics of AmB nano-assembly. Release kinetics of monomeric AmB from the as-synthesized nano-assembled AmB in phosphate buffer saline (PBS) at pH 7.4 at 37 °C. The AmB released at different time periods was evaluated at 408 nm using a UV-Vis spectrophotometer. Each point depicts an average of three estimations ± SD.

The biomimetically synthesized nano-assembled AmB was evaluated for the release of core AmB drugs under physiological conditions in PBS at pH 7.4 and temperature of 37 °C. Drug release kinetics was studied using the dialysis diffusion method for a time period of 250 h. The percentage release of drug versus time (in hours) was plotted as shown in Figure 2. The nanoassemblies showed a moderate release pattern over a period of 250 h. The release began with an immediate initial burst, and at the completion of 125 h, about 30 ± 1.9% of the AmB drug had been released into the PBS. It can be seen that about 46 ± 1.0% of the nano-assembled drug was released into PBS upon the completion of 250 h. The graph depicts the sustained release of AmB over an extended period of time.

The loading capacity for the as-formed AmB nanoassembly was calculated and it was found to be 78% of the monomeric form of AmB that was initially co-incubated with *Mangifera indica* extract.

### 3.4. Toxicity Assessment of the AmB-NA Formulation

#### 3.4.1. Assessment of Hemotoxic Effect Revealed Minimal Toxicity of AmB-NA on RBCs

**Figure 3:**
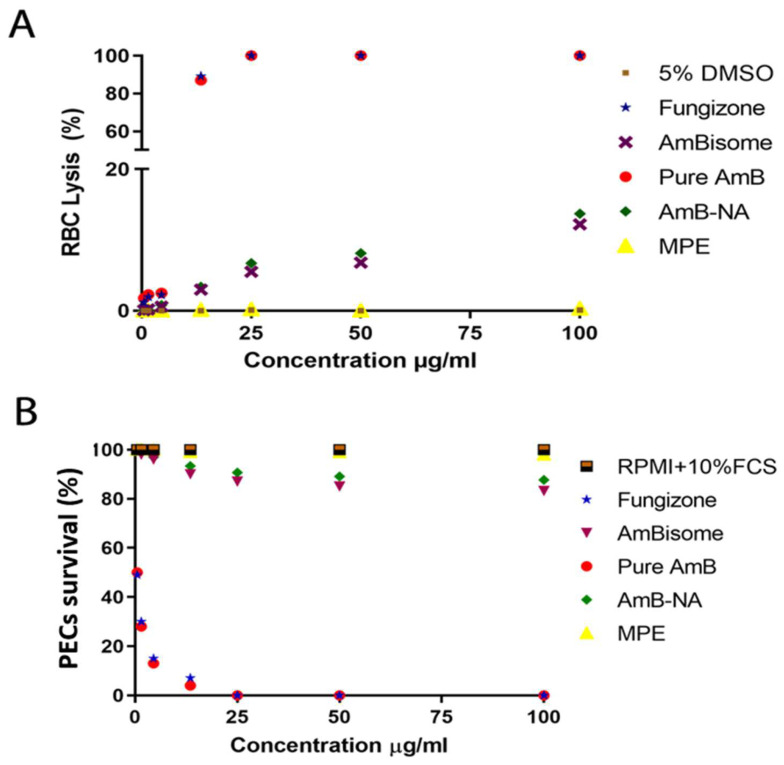
As-synthesized AmB-NA evoked minimal toxicity against living cells. (**A**) The extent of damage incurred to healthy RBCs upon exposure to AmB-NA was measured as a percentage lysis of total RBCs. Isolated RBCs (2 × 10^8^ cells/mL) were incubated with as-formed AmB-NA to assess the intrinsic toxicity of the later on animal cells. (**B**) Assessment of as-synthesized AmB-NA mediated toxicity on PECs-derived macrophages. The cells were exposed to AmB-NA for a period of 24 h. MTT assay was employed to assess the toxicity of as-synthesized AmB-NA; the extent of the killing of the treated cells was normalized to the control untreated cells. Data are reported as means ± SE of the quadruplet set of the experiment.

The RBC lysis assay was performed to compare the toxicity profile of AmB-NA with reference to other leishmanicidal formulations, including Fungizone™ and AmBisome as well as pure AmB. The extent of haemoglobin release as a measure of RBC lysis was illustrated as the percentage of total lysis (100%) induced by the exposure of cells to Triton X-100. The AmB-NA turned out to be the safest formulation at a concentration as low as 1 µg/mL with minimal haemolytic effects, whereas less than 15% haemolysis was observed at the concentration of 100 µg/mL of AmB-NA. AmBisome also displayed minimal haemolysis (maximum haemoglobin leakage was ~12.2 ± 0.91%) and served as a standard control. Moreover, 13.7 ± 2.4% haemolysis was evident for the AmBisome at a concentration of 100 µg/mL, which was almost similar to AmB-NA (Figure 3A). As expected, Fungizone™ was the most toxic formulation for the RBCs at all tested concentrations, and was thus taken as a positive control. It showed a sharp concentration-dependent increase in toxicity (2.23 ± 7.0% to 89.0 ± 5.3%) at a tested dose range (4.5 µg/mL to 13.5 µg/mL) and complete haemolysis at 25 µg/mL. The free form of AmB at 25 µg/mL or above induced toxicity to a similar extent as did Fungizone™.

#### 3.4.2. AmB-NA Were Negligibly Toxic to PECs-Derived Macrophages

The cytotoxic effect of biomimetic AmB-NA and common anti-leishmanial formulations and pure AmB were evaluated on PECs-derived macrophages. The cells treated with Fungizone™ and pure AmB showed only 7% and 4% survival percentages, respectively, at the dose of 4.5 and 13.5 µg/mL (Figure 3B). About ≥ 85% of macrophages survived even at high concentrations (100 µg/mL or above) of both the AmBisome and biomimetic nano-formulation (AmB-NA). The observation is apparent for the least toxic profile of the prepared biomimetic nano-formulation. The rank order of IC_50_ of the four formulations against the PECs in the ascending order was as follows: Fungizone™ (0.5µg/mL, 93% cytotoxicity at 4.5 µg/mL) ≥ pure AmB (0.5 µg/mL, 93% cytotoxicity at 4.5 µg/mL) ≥ AmBisome (>0.5 µg/mL; and 17% cytotoxicity at 100 µg/mL) ≥ AmB-NA (>0.5 µg/mL; 13% cytotoxicity at 100 µg/mL). The results demonstrated the superior safety profile of AmB-NA formulation tested against the reference anti-leishmanial drugs (pure AmB, AmBisome, and Fungizone™) as it exhibits the least cytotoxic effects amongst them all.

### 3.5. Killing Potential of AmB-NA on Leishmania-Infected PECs from BALB/c Mice

**Figure 4:**
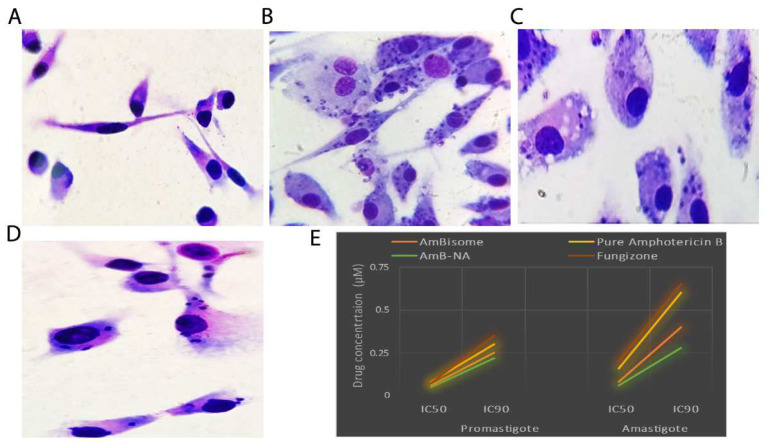
Parasiticidal potential of as-synthesized AmB-NA against *L. donovani*. The parasiticidal potential of the as-synthesized AmB-NA was determined on the basis of residual intracellular parasite load in peritoneal exudate macrophage in response to antileishmanial activity incurred by AmB-NA post 48 h of incubation with *L. donovani*. (**A**) Uninfected macrophages served as a negative control, (**B**) *L*. *donovani* infected macrophages, (**C**) Macrophages with reduced parasite load post-AmB-NA single-dose treatment, and (**D**) Macrophages representing a reduction in parasite load post-AmB-NA double dose treatment. (**E**) Graph demonstrating IC_50_ and IC_90_ for various AmB formulations against promastigote and amastigote. Fungizone-, pure Amphotericin B-, AmBisome-based formulations were included as the control.

The leishmanicidal effect of AmB-NA was assessed on *Leishmania*-infected PECs (Figure 4A–D). The biomimetic formulation (AmB-NA) was highly effective against both amastigotes and promastigotes of *Leishmania donovani*. IC_50_ and IC_90_ of AmB-NA for amastigotes were found out to be 0.06 µM and 0.28 µM, respectively (Figure 4E). Likewise, IC_50_ and IC_90_ of AmB-NA against promastigote were 0.05 µM and 0.22 µM, respectively (Figure 4E). Moreover, the parasite viability was also assessed against the reference leishmanicidal drugs (AmBisome, Fungizone™, and pure AmB) (Table 2). The IC_90_ of AmB-NA was found to be less than that of AmBisome (~1.5-fold), Fungizone™ (~2.3-fold), and pure Amphotericin B (~2-fold) (*p* < 0.05 for all three comparisons). Moreover, the CC_50_ value of pure AmB, Fungizone™, Ambisome, and Amb-NA in µM were 2.09, 1.808, 1.456, and 19.25, respectively, with a selectivity index of 12.56, 11.3, 18.2, and 19.25 (Table 2).

### 3.6. Effect of Drug on Leishmania Promastigotes

#### 3.6.1. AmB-NA Treatment Reduced Intracellular Thiol Content

**Figure 5:**
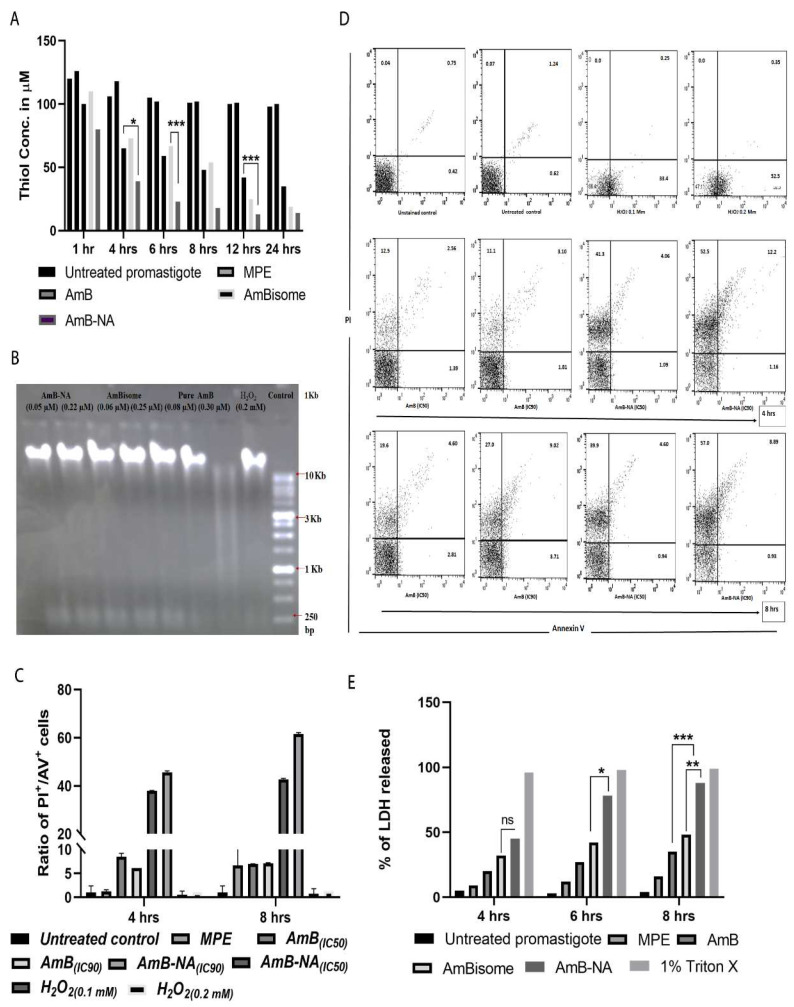
AmB-NA formulation-treated promastigote revealed reduced intracellular thiol and necrotic mode of cell death. (**A**) Effect of nanoparticles-based AmB-NA formulation as a function of the level of thiol derivative DTNB. Total intracellular reduced thiol was measured in deproteinized extracts of metacyclic *L*. *donovani* with or without drug formulation at the respective IC_50_ value of the drug formulation. After 12 h, AmB-NA (0.5 μM)-treated parasites revealed a ~3.2-fold decline in thiol release compared to AmB-treated parasites (*p* < 0.001). (**B**) Agarose (1%) gel electrophoresis of genomic DNA isolated from parasites treated with nanoparticles derived AmB-NA, AmB, AmBisome, and H_2_O_2_ with 100 bp and 1 kb DNA ladder as markers. Untreated and H_2_O_2_-treated (0.2 mM) parasites were taken as negative and positive controls, respectively. (**C**). A statistical representation of FACS data as a ratio of (%) of (PI+/AV+ cells) under similar conditions after 4 h and 8 h post-drug treatment. Promastigotes (1 × 10^6^ cells/mL) were treated with drugs at their IC_50_ and IC_90_ for 4 h and 8 h, respectively. Following 4 h incubation, the ratio was ~38 and ~8 for AmB-NA and AmB, respectively, whereas following 8 h incubation, it was ~42 and ~7, respectively. (**D**) Dot plot representation of FACS analysis corresponding to Annexin V and Propidium iodide-stained promastigotes after treatment with as-synthesized AmB-NA and commercially available AmB at their IC_50_ and IC_90_. (**E**) Evaluation of membrane leakage by LDH assay. A total of 1% Triton-X treated cells were measured as a positive control. Asterisks denote *p* values determined by one-way ANOVA analysis: *** *p* < 0.0001 compared with control, * *p* < 0.01 compared with the control. Analyzed data derived from at least 4 independent experiments * *p* < 0.05; ** 0.05 < *p* < 0.01; *** 0.01 < *p* < 0.001.

After 4, 6, 12, and 24 h, the thiol content in AmB-NA-treated (0.5 μM) parasites was found to be ~1.6, ~2.5, ~3.2, and ~2.5-fold lower, respectively, as compared to AmB-treated parasites (*p* < 0.001) (Figure 5A). However, the remarkable reduction of 2.9-fold was monitored in AmBisome-treated parasites compared to AmB-NA formulation.

#### 3.6.2. No Fragmentation (Apoptosis) of *L. donovani* DNA following Treatment with AmB-NA

DNA fragmentation was assessed to check apoptosis-associated DNA damage in *Leishmania* following AmB-NA, fungizone, or AmBisome treatment. No noteworthy DNA laddering (characteristic of DNA fragmentation) was detected in AmB-NA, AmBisome, or pure AmB-treated parasites (at IC_50_ and IC_90_), establishing the absence of apoptosis underneath these circumstances, whereas control (apoptotic) H_2_O_2_-treated cells revealed DNA laddering as anticipated (Figure 5B).

#### 3.6.3. Annexin/PI Assay Revealed Predominant Necrotic Cell Death in the Infected Host Macrophages Post AmB-NA Treatment

A necrotic manner of cell death was observed in Ld-infected PEC macrophages after treatment with AmB and AmB formulations (Figure 5C). The percentage of PI^+^ and AV^+^ promastigotes were taken as necrotic and apoptotic cells, respectively, following FACS analysis (Figure 5D). Therefore, an elevated ratio of the percentage of PI^+^/AV^+^ cells was an indicator of increased necrosis. At the 0.22 µM treatment condition, after 4h incubation, the ratio was ~38 and ~8 for AmB-NA and AmB, respectively, whereas, after 8h incubation, this ratio was ~42 and ~7. Hence, AmB-NA-mediated parasite killing is more necrotic than apoptotic.

#### 3.6.4. Estimation of LDH Membrane Escape in Treated Parasite Post AmB-NA Treatment

Extracellular release of LDH is associated with elevation in membrane damage resulting into necrosis. After 6 h incubation, at 0.5 μM concentration, released LDH was 2.7-fold and 1.8-fold more in AmB-NAtreated parasites compared to AmB- and AmBisome-treated cells, respectively (Figure 4E, *p* < 0.001). Fascinatingly, under an analogous treatment regime, the variation in LDH release was insignificant (2.3-fold and 1.6-fold for AmB and AmBisome, respectively, Figure 5E). H_2_O_2_, positive control treatment causes absolute cell damage. Therefore, AmB-NA is more necrotic than AmB and AmBisome against *L. donovani* promastigote.

#### 3.6.5. AmB-NA Treatment Induces Mitochondrial Membrane Depolarization

**Figure 6:**
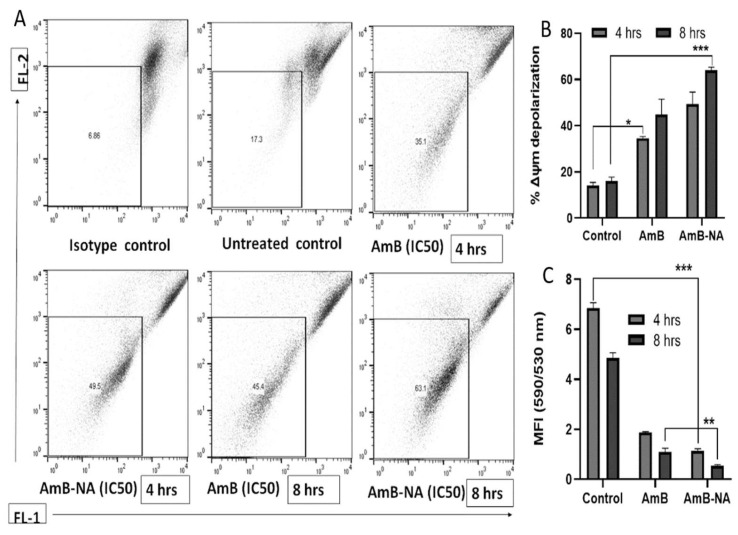
Effect of AmB-NA on the mitochondrial membrane potential of *L. donovani* promastigotes. The effect of AMB-NA formulation on *L. donovani promastigotes* mitochondrial membrane was determined. A known population of *L. donovani* promastigotes (1 × 10^6^/mL) was treated with as-synthesized AmB-NA for 4–8 h at 24 °C. The AmB-NA treated parasites were stained with JC-1 as described in the method. (**A**) FACS analysis of JC-1 mitochondrial potential marker staining 4 h and 8 h post-exposure with AmB-NA formulation. Panels represent density plots from a single analysis. Δψm depolarization monitored in the upper panel correspond to cells with intact mitochondrial membrane, whereas cells gated in the lower panel depicted damaged membrane of the treated cells with a loss of Δψm. (**B**) Graphic representation of mean values of the fluorescence corresponding to the mitochondrial membrane potential of the treated cells (Δψm collapse ± SD). (**C**) MFI was calculated employing a ratio of fluorescence at 590/530 nm. Asterisks (*) denote *p* values determined by one-way ANOVA analysis: *** *p* < 0.0001 compared with control, ** *p* < 0.001 compared with control, * *p* < 0.01 compared with the control. Analyzed data derived from at least 4 independent experiments.

As mitochondria play a vital role in energy production and, thus, survival of the *Leishmania* species, the parasite’s mitochondrial membrane potential (Δψ_m_) was evaluated using the flow cytometry technique [46]. The Δψ_m_ exhibited a substantial level of depolarization in AmB-NA, i.e., ~1.5 and 4.2-fold, in comparison to AmB and control after 8 h incubation (Figure 6A,B). As JC-1 fluoresces red in live cells and green in damaged cells, the decrease in Δψ_m_ is reflected as a fall in the red/green (590/530) ratio or J-aggregates/J-monomers ratio. Following 8 h incubation, there was a substantial decrease in the relative ψ_m_ of *Leishmania* parasites following treatment with AmB (1.09 ± 0.15) and AmB-NA (0.55 ± 0.04, *p* < 0.001), in comparison to the control (4.85 ± 1.21), indicating mitochondrial membrane depolarization in treated parasites (Figure 6C).

### 3.7. Assessment of Biochemical Parameters in a Mouse VL Model

The analysis of biochemical parameters (transaminases, ALT [alanine transaminase] and AST [aspartate transaminase]; blood urea nitrogen, BUN; serum creatinine) pertaining to the kidney and liver health of mice was also done. The VL-infected mice treated with Fungizone™ and AmB-NA did not exhibit any significant difference in the levels of transaminases (ALT and AST) as compared to the control. However, an AmB-NA concentration of 10 mg/kg body weight or above altered the biochemical markers of liver and kidney health, although the levels were up to the AmBisome-treated group. The AmB-NA-treated mice group showed an exceptional reduction in splenomegaly (from 7.8 mm to 0.75 mm) and hepatomegaly (from 4.3 mm to 0.88 mm) and gain in body weight parallel to the healthy controls. Mice treated with Fungizone™ showed significantly upregulated BUN and serum creatinine concentrations (Table 3). Furthermore, parameters such as WBC count, haemoglobin content, lymphocytes, and magnitudes of sodium and potassium were also evaluated in mice before and after treatment with AmB-NA and other reference drugs (AmB, AmBisome, and Fungizone™) to have a comparative estimate of the effect of treatment on VL.

### 3.8. DTH Response Post-Treatment with AmB-NA

**Figure 7:**
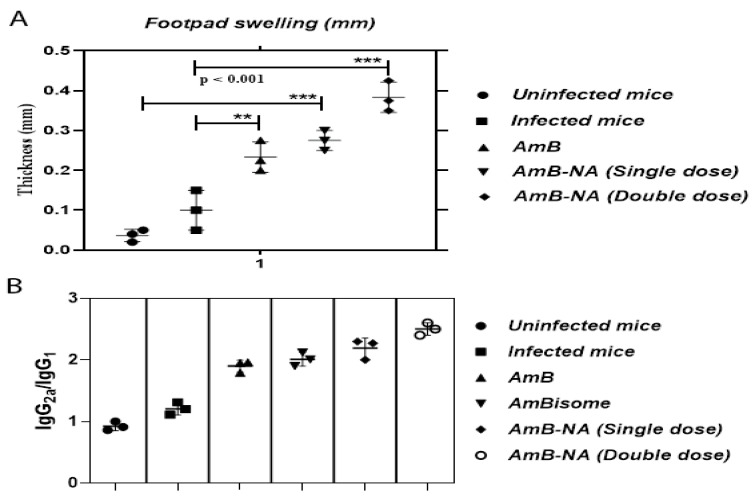
Immunomodulatory potential of the AmB-NA in the L. donovani-infected BALB/c mice. The *L. donovani*-infected animals were treated with various as-synthesized AmB-NA formulations following a published protocol described in the method. The splenocytes of the cured animals were isolated after 4-week post-treatment as described in the method. (**A**) DTH response evoked in the immunized animal post-treatment with various forms of AmB. Footpad swelling was considered as a parameter to determine the as-generated cell-mediated immune response in the treated BALB/c mice. The treatments (50 μg SLA) were applied in a footpad, and the magnitude of DTH was assessed post 48 h. SLA was inoculated in the uninfected group, infected, untreated, and variously treated groups (AmB-NA (single dose), AmB-NA (double dose), and AmB). (**B**) Four-week post-treatment, blood from various experimental groups was collected to separate sera. ELISA was performed to evaluate antibody levels. IgG2a/IgG1 isotype ratios for AmB and AmB-NA single dose-treated mice were 1.9 and 2.1, respectively, thus proving AmB-NA to have better therapeutic efficacy against VL. Data represented means ± SE for mice per group and are representative of three independent experiments with similar results. ** *p* < 0.01, *** *p* < 0.001.

Chemotherapeutic intervention and a successful cure resulting from anti-leishmanial therapy are chiefly associated with DTH response (an index of cellular immunity), and therefore, classical cell-mediated immunity [52,58,59]. DTH response was evaluated in normal, infected, and treated mice (Figure 7A). Recovery from infection after AmB and AmB-NA (single and double dose) treatment was conveyed by the generation of a strong DTH response (*p* < 0.001). After 48 h, the average thickness of the footpad ranged from 0.02 mm to 0.45 mm in various study groups. Swelling increased by 2.7-and 3.8- fold in the case of AmB-NA single and double-dose administered mice, respectively, in comparison to infected mice, whereas it was 2.3-fold for AmB. Hence, a substantial difference in footpad swelling was monitored in AmB-NA-treated mice compared to untreated mice.

### 3.9. AmB-NA-Treated Mice Induced Higher IgG_2_a to IgG_1_ Ratio

To examine the Th_1_/Th_2_ cross-regulation and to decipher the involved protective mechanism, we measured the serum levels of parasite-specific IgG isotypes by indirect ELISA. Preceding studies established that the level of IgG_2_a reliant on IFN-γ, whereas the level of IgG_1_ correlates to that of IL-4 secretion. Thus, the ratio of IgG_2a_/IgG_1_ was used as a surrogate marker for Th_1_/Th_2_ responses [59]. Interestingly, the mice treated with an AmB-NA single and double dose displayed significantly more abundance of IgG_2a_ antibodies as compared to IgG_1_ post-treatment (*p* < 0.005) (Figure 7B). The ratio of IgG_2a_ to IgG_1_ was found to be 1.9, 2.0, 2.1, and 2.53 in AmB, AmBisome, AmB-NA single, and double-dose-treated mice, respectively. The results suggested a correlation between humoral immune response and disease control. The isotype data analysis revealed that AmB-NA is a better drug in comparison to all the existing drugs in trends against VL.

### 3.10. Parasite Burdens in Vital Organs of VL-Challenged Mice

**Figure 8:**
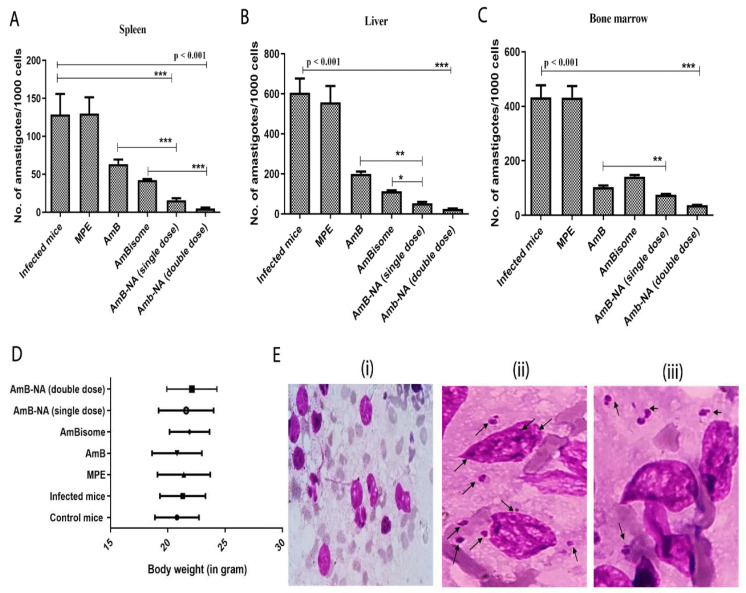
As-synthesized AmB-NA treatment resulted in successful clearance of the in vivo parasite burden. The treatment with AmB-NA resulted in the killing of the *L. donovani* systemic infection present in the experimental mice’s organs such as the spleen, liver, bone marrow, etc. Infected mice with no AmB treatment were considered as controls. The parasite burden in the spleen (**A**), liver (**B**), and bone marrow (**C**) was determined microscopically (Giemsa-stained cell smear) and expressed as no. of amastigotes/1000 cells. Data represented means ± SE for mice per group and are representative of three independent experiments with similar results. * *p* < 0.05, ** *p* < 0.01, *** *p* < 0.001, (**D**) The relative body weight of the experimental mice before and after drug administration. (**E**) Giemsa-stained uninfected (**i**), *L. donovani*-infected (**ii**) and *L. donovani* infected AmB-NA treated cells (**iii**). The arrows are showing the Ld bodies. Data represent the means ±SE for six animals per group. Results correspond to one of the three similar experiments.

To determine the efficacy of AmB-NA treatment, a comparative evaluation of parasite load in the spleen, liver, and bone marrow of the experimental animals was undertaken. We also examined corresponding body weight changes in the mice belonging to various experimental groups. There was a significant decrease in parasite load in the spleen, liver, and bone marrow in the group of mice treated with AmB-NA (*p* < 0.005), in comparison to positive control groups (Figure 8A–C). The microscopic examination of the spleen of the infected mice demonstrated maximum clearance of parasites in the group treated with a single dose as well as a double dose of AmB-NA, i.e., 16 ± 5 and 5 ± 2 amastigotes per 1000 spleen cells, respectively, whereas 129 ± 47, 64 ± 11, and 42 ± 3 amastigotes per spleen cells were observed in mice treated with the untreated control and pure AmB and AmBisome, respectively. Likewise, 600 ± 123, 200 ± 20, 113 ± 8, 53 ± 11, and 25 ± 3 amastigotes/1000 hepatocytes were observed, respectively, in the untreated group of mice, and groups treated with AmB, AmBisome, AmB-NA single dose, and AmB-NA double dose. Moreover, bone marrow burden revealed 75 ± 6 and 37 ± 2 amastigotes/1000 bone marrow cells, respectively, in mice administered with a single and double dose of AmB-NA compared to 141 ± 10 amastigotes following AmBisome treatment. An increase in the body weight in the experimental mice was corroborated by evident splenomegaly and hepatomegaly. Moreover, a significant increase in weight equivalent to healthy mice was noticed in mice after AmB-NA double dose administration (Figure 8D). Giemsa-stained microscopic examination detected Ld bodies inside splenocytes and hepatocytes of infected mice (Figure 8E).

### 3.11. Quantitative Analysis of T-Helper Type (Th_1_/Th_2_) Cytokines 

**Figure 9:**
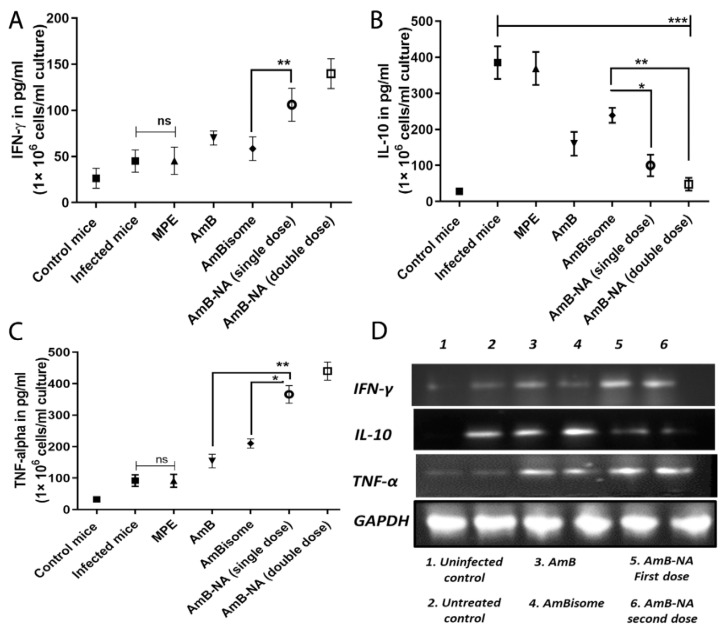
Assessment of the pro-inflammatory potential of AmB-NA against murine visceral leishmaniasis. Four-week post-treatment splenocytes from different study groups (uninfected, infected, and various drug-treated BALB/c mice) were cultured and incubated in a CO_2_ incubator in the presence of 48 h SLA stimulation at 37 °C. Cell-free supernatant was used to evaluate cytokine profile through ELISA (**A**) IFN-γ, (**B**) IL-10, and (**C**). TNF-∝ (Data represented as means ± SE for six mice per group and are representative of two independent experiments with similar results. * *p* < 0.05, ** *p* < 0.01, *** *p* < 0.001), ns = non-significant, and (**D**) Semiquantitative PCR for genes specific for mice IFN-γ, IL-12, IL-10, and GAPDH.

We performed a range of tests to establish the anti-leishmanicidal activity of the as-synthesized AmB-NA. Hence, post-treatment cellular immunity accountable for the establishment of prophylaxis was assessed. The cytokines have a leading role in activating and shaping the cellular adaptive immunity and macrophage effector functioning to eliminate intracellular *Leishmania* amastigotes. The strategy was to ensure whether these formulations could induce or suppress cytokine production levels post-treatment. Therefore, we have quantitively evaluated some key cytokines (IL-10, IFN-γ, and TNF-α) in treated and untreated mice groups (Figure 9A–C) using ELISA. The production of IFN-γ increased by ~2.3, ~3.1, and ~1.5-fold after treatment with AmB-NA single dose, AmB-NA double dose, and pure AmB, respectively. Additionally, the TNF-α level was found to be ~2.3, ~1.7, and ~3.9-fold raised in AmB-NA single-dose-treated mice in comparison to AmBisome, AmB-NA-treated, and infected mice, respectively, (*p < 0.005*), whereas the production of IL-10 was down-regulated by ~3.85, ~8.0, ~2.4, and ~1.6-fold by AmB-NA single dose, AmB-NA double dose, AmB, and AmBisome, respectively, as shown in Figure 6. The observed results suggest that the biomimetic nanoaggregates of AmB enhanced cytokines production to induce a Th_1_ biased immune response to debar intracellular infection of *Leishmania*. The expression level of various cytokines was further validated by employing an RT-PCR (Figure 9D).

### 3.12. AmB-NA Administration Increases NO and ROS Production

**Figure 10:**
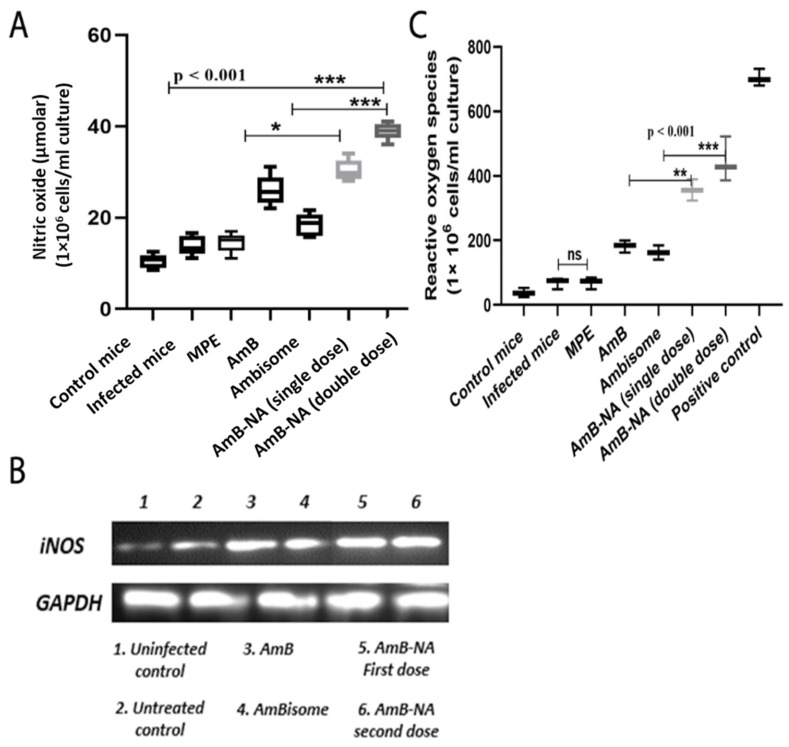
Assessment of NO generation. The figure denotes nitric oxide (NO) released from macrophages belonging to various groups of untreated and treated mice (**A**) Quantitative estimation of NO produced (µM) in the cultured supernatant of the splenocytes. The freshly isolated mononuclear splenocytes cells were plated in a 12-well microtiter culture plate (1 × 10^6^ cells/mL/well) and incubated in the CO_2_ incubator at 37 °C for 48 h. Data represented means ± SE for six mice per group and are representative of three independent experiments with similar results. * *p* < 0.05, ** *p* < 0.01, *** *p* < 0.001, ns = non-significant. (**B**) Semi-quantitative PCR for genes specific for iNOS, GAPDH was used as a control. (**C**) Measurement of reactive oxygen species (ROS). Data represented means ± SE for six mice per group and are representative of three independent experiments with similar results. * *p* < 0.05, ** *p* < 0.01, *** *p* < 0.001.

NO is the crucial effector molecule that helps in the killIng of the *Leishmania* parasite and is majorly produced by IFN-γ stimulated classical macrophages. To check the influence of AmB-NA treatment on NO levels and its role in *L. donovani* killing, NO level was determined in splenic mononuclear cell culture supernatants. In the case of AmB-NA- treated *L. donovani* challenged mice, we found a three-fold higher level of nitrite (corresponding to NO) production in comparison with infected, untreated controls (Figure 10A). The AmB-NA formulation induced significantly higher NO production (*p* < 0.0001). Moreover, RT-PCR further validated the up-regulated expression of iNOS in the differentially treated mice (Figure 10B). In the case of AmB-NA-treated, *L. donovani*-challenged mice, we found a 1.96, 2.1, 5.2-fold higher level of ROS production compared with AmB, AmBisome, and infected untreated controls, respectively (Figure 10C). The upregulated expression of NO is commensurate with elevated IFN-γ produced from the splenocytes of the AmB-NA treated mice (Figure 9).

## 4. Discussion

Despite continuous efforts of the scientific community across the world, no commercial vaccine has been developed against VL to date [60,61]. The prevailing situation compels us to rely on chemotherapeutic approaches to control this life-threatening disease. Unfortunately, the chemotherapeutic-based approaches suffer from grave impediments such as the constant evolvement of drug-resistant *Leishmania* isolates, compromising efficacy, toxicity, prolonged courses, and parenteral routes of administration of most of the available anti-leishmanial drugs [62]. It is, thus, decisive that efforts for discovering new drugs or repurposing old ones are essential for effective leishmaniasis chemotherapy.

Systemic intravenous administration of AmB has remained a very effective therapy against VL with a cure rate of >95% [63]. Nevertheless, the unfavorable physicochemical properties of the free form of AmB and associated chronic nephrotoxicity or acute infusion-related toxicities restrict its efficacy [11,64]. The micellar formulation, Fungizone^®^, rapidly releases AmB in the plasma and renders toxicity to mammalian cells [65]. This has become one of the most critical grounds for its rejection. 

In the recent past, several attempts have been diverted toward the development of a novel drug delivery system to improve the therapeutic index of AmB [21]. Amongst the various formulations, AmBisome is the most acceptable liposomal preparation with a lower toxicity profile, sustainable release, desirable pharmacokinetics or pharmacodynamics, prolonged circulation time, and targeted delivery of core drug at the site of infection [12,66]. Unfortunately, the exchange of lipid moiety between liposomes and serum lipoprotein/bio-membranes leads to compromised stability that ensues in the loss of the encapsulated content [67]. Moreover, the requirement for a cold chain [68], high production and import cost [69,70], complex production process [71], the occurrence of acute kidney injury [72], development of post-Kala-Azar dermal leishmaniasis after apparent cure [73], and reports of VL relapses are some of the shortcomings of AmBisome formulation, limiting its utilization throughout the globe. 

The AmB-mediated toxicity has been studied extensively. It is widely reported that higher-order aggregates of AmB display greater reduced toxicity than oligomeric small water-soluble aggregates [21,74,75]. Moreover, the super-aggregated form of AmB demonstrated better anti-leishmanial [76], anti-plasmodial [77], and antifungal activities [21]. It is tempting to speculate that the toxicity of AmB can be meticulously controlled by tweaking its degree of aggregation. 

Recently, we exploited plant leaf extract for green synthesis of anti-cancerous [32] as well as antifungal agents [21]. The biomimetic synthesis could have significant implications as nano-assembled forms of a drug exhibit superior properties than the core drug. The nanocrystal-based formulation is also devoid of excipient-related constraints [78]. Therefore, we envisaged that the extract from other plant sources would also induce the formation of organic nanostructures under similar conditions. Thus, we incubated AmB with mango fruit pulp extract in a manner analogous to our previous study [21] to mediate the bio-fabrication of AmB nano-aggregates.

UV-Vis spectroscopic studies were performed to ascertain the formation of AmB nano-assemblies. The UV-Vis spectra of AmB are dependent on its aggregation state. The AmB monomers exhibit maximum absorption (λ_max_) at 405–409 nm, whereas the dimer or oligomeric form displays an absorption peak at 340–360 nm. For the super-aggregated form, the absorption maxima shift to wavelengths lower than 322 nm [79]. The nanostructures demonstrated spectra typical of AmB super-aggregates, as indicated by a UV spectral shift of the maximum absorption to 320 nm (Figure 1). For AmB, absorption peaks at a lower wavelength that has been reported for AmB-deoxycholate complex [80], AmB nano-aggregates [21], heat-induced super aggregates [79], as well as for AmBisome [81,82].

Absorption at a shorter wavelength implies tight packing of nonpolar polyene chains, with neighboring interacting molecules consisting of a roughly equiprobable population of parallel or antiparallel AmB dimers that rapidly interconvert into each other [83]. It can be stipulated that the AmB dimers/tetramers act as seeding points (nucleus) for the condensation of monomers and eventually lead to the formation of super-aggregated NAs. The mango fruit pulp extract contents function both as a driving force as well as a stabilizing agent for the whole process. Thus, the mixing of AmB with pulp extract leads to the formation of precise nanostructures (super-aggregates) with characteristic spectral properties. Moreover, FTIR data (Figure 1E) suggest that the assemblage of drug molecules in nano assemblies does not alter its basic chemical entity. It can be inferred that the parent structure of the AmB molecule is retained during the synthesis of AmB-NA. 

The as-synthesized AmB-NA formulation was further characterized regarding the particles’ size, shape, and zeta potential. The size obtained by TEM was found to be lesser than the aggregate diameter established by DLS (Figure 1). Different methods of size determination may not be exactly complementary. TEM provides information about the size and shape of individual nanoparticles dried under a high vacuum, whereas DLS measures diffusion in particle dispersions, which can be interpreted using the Stokes-Einstein equation to yield an ensemble average hydrodynamic particle diameter. Hence, there is a discrepancy between TEM and DLS-based size determination [84]. High zeta potential (either positive or negative), more than 30 mV, maintains a stable system. Extremely positive or negative zeta potential values cause larger repulsive forces; this repulsion between similarly charged particles prevents aggregation of the particles and thus ensures easy redispersion [85]. Therefore, it can be said that AmB-NA prepared using pulp extract are quite stable.

AmB-nano-assemblies were further evaluated for their toxicity and anti-leishmanial activity. The method for the determination of toxicity of AmB formulations involved in in vitro incubations of formulations with red blood cells and animal lethality tests in mice are the most commonly used methods. The commercial formulation of AmB, i.e., Fungizone™, is notorious for eryptosis. Hemotoxicity is elicited by means of cell shrinkage and cell membrane scrambling, ensuing augmented calcium ions into erythrocytes, and finally leakage of intracellular constituents such as potassium or hemoglobin from RBC. Therefore, the membrane damage triggered by AmB-NA, reference control formulations, and in vitro haemolysis was assessed in support of providing a dependable measure for reckoning the membrane injury caused in vivo. There was approximately 90% haemolysis after a 24 h incubation of Fungizone™ and pure AmB solutions (13.5 µg/mL), whereas at the same concentration, AmB-NA-treated RBCs revealed only 3.38% eryptosis (Figure 3A). This is consistent with the findings of Asthana et al., who reported 100% and 5.5% haemolysis at a concentration of 25 µg/mL [86]. In agreement with the current study, aggregated AmB-NA encrypted its reduced RBC haemolytic activity compared with Fungizone™ [79].

The cytotoxic effect of AmB-NA on the survival of PECs demonstrated the least amount of toxicity among all the study groups, with only 12.3% cytotoxicity at 100 µg/µL; i.e., more than 85% of macrophages survived even at a higher dose of more than 100 µg/µL of as-synthesized AmB-NA. In agreement with the previous study, AmB-NA exhibit substantially reduced toxicity against the PECs compared to Fungizone™ and pure AmB, which displayed dose-dependent toxicity (Figure 3B). Coherent to RBC lysis results, PEM cytotoxicity data advocated that the super-aggregated biomimetic nature of AmB-NA might be responsible for its enormously less toxicity toward living cells. Hence, the as-synthesized AmB-NA formulation fulfills one of the most critical benchmarks for being an effective therapeutic agent.

The low toxicity of AmB-NA can be attributed to the presence of a super-aggregated form of the core drug. AmB inside the AmB-NA acts as a reservoir of monomeric AmB and releases it in a slow and sustained manner, such that the drug is not readily available to bind to the host cell plasma membrane, thereby exerting reduced toxicity. In the case of Fungizone, the drug is released in a burst phase, making the total administered dose of AmB available abruptly, resulting in more apparent toxic side effects. Moreover, the slow release of the drug from the AmB-NA formulation ensures that the drug remains a monomer and does not form oligomeric aggregates, which is the species mainly responsible for drug toxicity.

The anti-leishmanial potency of promastigote was noteworthy in the case of AmB-NA, being ~1.4 and ~1.6-fold higher than that of Fungizone™ and AmB, respectively. This result was consistent with the findings of Carrol and colleagues [87]. Evaluation of the anti-leishmanial efficacy of the biomimetic formulation revealed good anti-leishmanial activity against intra-macrophage amastigotes. The 50% parasite growth-inhibitory efficiency of the AmB-NA formulation for the amastigotes was ~2.3-fold better than Fungizone™. It is interesting to note that the as-synthesized AmB-NA formulation is noticeably more effective than both free AmB as well as its liposomal formulation, AmBisome. The substantially enhanced efficacy and reduced toxicity are concordant with the findings of previous AmB formulations [86,88]. Moreover, our findings are in concordance with the previous finding reporting the significant anti-leishmanial activity of *Mangifera indica* extracts against *Leishmania donovani* promastigotes in vitro and exploiting the MTT assay [36].

In a manner similar to the observed AmB-NA efficacy, the previous studies too reported that promastigotes are more susceptible to AmB treatment as compared to intra-macrophage amastigotes (PEC) [48,87,89]. Overall, it was demonstrated that amongst commercially available AmB formulations, Fungizone™ is the most toxic, and AmBisome™ is the least toxic. Hence, AmB-NA formulation demonstrated the least amount of toxicity and higher IC_50_ among all. The higher IC_50_ indicates less potassium leakage and, therefore, lower toxicity for RBCs [21,89]. The selective activity of AmB-NA against fungi and *Leishmania* instead of mammalian cells is because of its higher affinity for ergosterol and episterol, which are found in parasite membranes, more so than for cholesterol, which is the mammalian sterol [90].

The intracellular-reduced thiol of *Leishmania*, a component of trypanothione, plays a primary role in maintaining the redox homeostasis of parasites. We found that the AmB-NA downregulated intracellular reduced thiol content more significantly than those other formulations (Figure 5A). This indirectly suggested an induction in oxidative stress that may lead to the death of the parasite. No DNA fragmentation post-AmB-NA treatment suggested a necrotic mode of killing (Figure 5B). The observation agrees with the increased ratio of the percentage of PI^−^/AV^+^ cells post-treatment with as-synthesized AmB-NA formulation (Figure 5C) and elevated LDH level (Figure 5E).

Mitochondria play an important function in the survival and death of cells [90,91]. As *Leishmania* has only one big mitochondrion that fulfills its energy (ATP) needs, it can be represented as a potent target for the drug. We found that AmB-NA could cause a significant reduction in mitochondrial membrane potential along with structural and physical damage to mitochondria (Figure 6). The permeability of the mitochondrial outer membrane is crucial for mitochondrial membrane integrity, and if compromised, may ensued in cell death [92]. Overall, these findings suggest that in comparison to free AmB, AmB-NA induces elevated and severe mitochondrial dysfunction in *L. donovani* promastigotes by triggering depolarization of mitochondrial membrane potential, loss of mitochondrial integrity, and depletion of mitochondrial integrity of the cellular ATP pool.

The desirable in vitro and ex vivo features of as-synthesized AmB-NA led us to further assess its in vivo anti-leishmanial activity against the VL parasite. In the visceral form of leishmaniasis, parasites live and propagate within the phagolysosome of infected macrophages of the spleen and liver [93]. Hence for the determination of in vivo antileishmanial activity, the parasite load in BALB/c mice (treated with 5 mg/kg b.w AmB-NA or AmBisome and 1 mg/kg b.w AmB) was determined under the treatment regimen employed, and AmB-NA elicited a marked improvement over a pure form of AmB and similar results to that of AmBisome in terms of parasite burden and survival. Moreover, mice show increased body weight and the least amount of parasite burden after treatment under a double-dose regime. A significant reduction in the parasite burden was noticed in the case of AmB-NA as compared to AmBisome. This is in concordance with earlier reports [59,86].

Moreover, splenomegaly and hepatomegaly scores were also subdued post AmB-NA treatment. The liver functioning study suggested that AST and ALT levels returned to the values of those of uninfected healthy mice post treatment with AmB-NA. Interestingly, a double-dose regimen revealed more favorable hematological and biochemical parameters. 

DTH is considered as a predictor of cell-mediated immunity elicitation. The production of a robust DTH response post-AmB-NA treatment characterizes recovery from infection (Figure 7A). Moreover, to understand the immunomodulatory effect of AmB-NA formulation, we measured the level of total IgG, IgG_1_, and IgG_2a_ against *L. donovani* in the serum of mice treated either with AmB-NA and AmB compared with mice without treatment. The highest IgG_2a_/IgG_1_ ratio in mice treated with AmB-NA formulation, which controlled the progress of infection for *L. donovani*, redirects the immune response caused by the infection stemming from a Th_1_-type immune response (Figure 7B). This explains the elevated levels of pro-inflammatory cytokines obtained in this group as compared to other groups, as this response is considered most suitable for the control of infection.

Evidence suggested that defense against *Leishmania* is concomitant with a specific immune response switch (Th_2_-Th_1_ paradigm) for complete parasite clearance [48]. Accumulating evidence also suggested that the immunomodulatory function of AmB is coherent with its antileishmanial accomplishments (Figure 9) [94]. AmB-treated animals were in parallel with elevated Th_1_ (TNF-alpha, IFN-γ) and reduced Th_2_ (IL-10) cytokines. Interestingly, because of parasite clearance and cures, the upregulated IFN-γ and TNF-α concentrations and downregulated IL-10 levels were demonstrated in mice splenocytes (Figure 9). A similar result validating the inherent immunomodulatory activity of AmB [95,96] and *Mangifera indica* nano-particles induced anti-inflammatory, anti-trypanosoma, and immunomodulatory anti-leishmanial activity was reported earlier [35,36,37] AmB-NA single-dose and double-dose-administered mice exhibited 2.3 and 3.1-fold enhanced concentrations of IFN-gamma in splenocytes of treated mice. Although the course of disease progression ensuing *L. donovani* infection is still restrained by the cytokine network and an array of T cell responses [93,97], it is well established that the major performer in the disease pathology of VL is IL-10, which inactivates macrophages to initiate protective signals and nitric oxide production, leads to the obstruction amastigotes killing [98]. The current study measured noteworthy suppression (3.85 and ~8-fold) of IL-10 secretion after treatment with single and double-dose of AmB-NA, respectively. Therefore, the immunomodulatory effect of AmB-NA could be a key factor for therapeutic success against experimental VL. Oxidative stress-mediated toxicity was reported for the AmB-NA induced release of nitric oxide. Moreover, proportionately enhanced nitric oxide and ROS production established evidence of the Th_1_ paradigm following AmB-NA (Figure 10). Moreover, a similar pattern of results was obtained by semi-quantitative RT-PCR, which further strengthened our findings.

The AmB-NA formulation was effective in killing the parasite in experimental animals more proficiently than other commercial AmB formulations. The present study revealed a 4.04, 3.77, and 1.37-fold reduction in parasite burden in the spleen, liver, and bone marrow, respectively, post AmB-NA single-dose treatment in comparison to AmB, whereas a reduction in case of the double dose was 7.63, 5.12, and 1.54-fold, respectively. Similar consequences were demonstrated by researchers who showed that heat-treated Fungizone™ showed approximately two-fold increased anti-leishmanial activity over that of the untreated formulation in vivo [76]. This might be the factor behind super aggregate nano-assemblies of AmB-NA on reaching the site of infection and killing parasites more proficiently compared to other groups. Therefore, the AmB-NA formulation of AmB may replace available formulations of AmB in the future, and formulations of new AmB-nano aggregates with other antileishmanial agents may provide new visions for drug delivery.

## 5. Conclusions

Amphotericin B (AmB), a polyene class-based antibiotic, has extensively been put into use for the treatment of leishmaniasis and several types of fungal infections. Various lipid-based formulations of the antibiotic have been introduced to overcome the intrinsic toxicity issues of this important drug. However, the toxicity of the employed lipids used in the development of nano- formulations, and other associated concerns pose their own kind of shortcomings of the AmB. In this paper, we have presented a novel idea for improvement in the efficacy of AmB formulation by employing a green synthesis approach to develop nanosized particle aggregates of this important drug. The as-fabricated nano-assembled AmB formulations have presented us with fetching physicochemical properties, sustained drug release, and negligible toxicity constraints. The nano-assembled formulation is effective against both amastigotes and promastigotes of *Leishmania donovani*. Annexin/PI assay has divulged the necrotic mode of cell death in the infected host macrophages. The excipient-free AmB nano-aggregated formulation significantly reduced the parasite load in the spleen, liver, and bone marrow of mice as compared to AmB or AmBisome treatment alone. The as-developed nano-formulation of AmB induced three-fold higher levels of NO and ROS in comparison to untreated infected controls. Furthermore, RT-PCR results have confirmed the up-regulation of iNOS gene expression upon the administration of as-synthesized nano-assemblies of AmB.

## Figures and Tables

**Table 1:** Particle size and zeta potential of AmB-NA.

Sample	Average Zeta Diameter (nm)	Zeta Potential (mV)	Z-Average ^a^ (nm)	PDI	Aqueous Solubility (mg AmB/mL)	Loading Capacity (%)
**AmB-NA**	96 ± 0.8	−34.2 ± 0.3	101 ± 0.7	0.22 ± 0.00	1.0	78

**Note:**^a^ Z-average is the intensity weighted hydrodynamic size of a collection of particles measured by dynamic light scattering. Abbreviations: PDI, polydispersity index.

**Table 2:** In vitro antileishmanial activities (IC_50_ and IC_90_) of AmB-NA compared to those of Fungizone and AmBisome in macrophages infected with *L. donovani* amastigotes, observed after 48 h of incubation. CC_50_ was estimated after 48 h of drug treatment to macrophages. The SI represent the selectivity index calculated by dividing the CC_50_ by the IC_50_ value obtained after treatment of cultured macrophages after 48 h.

DRUGS	PROMASTIGOTE		AMASTIGOTES		
	IC_50_	IC_90_	IC_50_	IC_90_	CC_50_–SI
**AMBISOME**	0.06 µM	0.25 µM	0.08 µM	0.5 µM	1.5–18.2
**PURE AMB**	0.08 µM	0.3 µM	0.16 µM	0.9 µM	2.0–12.5
**AMB-NA**	0.05 µM	0.2 µM	0.06 µM	0.4 µM	1.2–19.3
**FUNGIZONE**	0.08 µM	0.3 µM	0.16 µM	0.8 µM	1.8–11.3

**Table 3:** Clinical symptoms, hematological as well as biochemical parameters of control and infected mice before and after anti-leishmanial therapy.

Study Groups	Healthy (*n* = 5)	Infected Mice (*n* = 13)				
		Before Treatment	After Treatment			
	Balb/c (*n* = 5)	Balb/c (*n* = 3) AmB	Balb/c (*n* = 3) AmB	(*n* = 3) Ambisome	(*n* = 3) AmB-NA (SD)	(*n* = 3) AmB-NA (DD)
Age (weeks)	12–16	12–16	12–16	12–16	12–16	12–16
Weight (g)	25–35	25–35	25–35	25–35	25–35	25–35
Body Temperature (in °F)	97.5 ± 0.5	100.56 ± 1.89	97.76 ± 1.83	97.22 ± 0.375	97.56 ± 1.83	97.4 ± 0.35
Hepatomegaly (in mm)	0	4.3 ± 2.26	1.8 ± 1.76	0.9 ± 0.94	0.88 ± 0.72	0.69 ± 0.82
Splenomegaly (in mm)	0	7.8 ± 3.39	2.9 ± 2.99	0.8 ± 1.13	0.75 ± 1.12	0.71 ± 1.03
Haemoglobins (g/dl)	14.76 ± 0.95	9 ± 1.44	10.8 ± 1.84	14.11 ± 0.89	13.5 ± 1.64	14.2 ± 0.69
WBC (white blood cells) (per mm^2^)	6760 ± 1581.37	9850 ± 949.10	6715 ± 1045.10	5809 ± 486.85	5909 ± 386.75	5509 ± 687.15
Lymphocytes (%)	32.46 ± 5.73	46.5 ± 8.19	29.5 ± 6.5	25.6 ± 2.54	24.4 ± 2.28	24.2 ± 2.74
AST (U/L)	150.1 ± 14.35	235.9 ± 16.01	180.2 ± 13.93	150.6 ± 18.60	170.1 ± 12.60	155.4 ± 17.12
ALT (U/L)	90.05 ± 10.58	125.12 ± 13.73	129.96 ± 14.53	102.23 ± 9.28	98.45 ± 9.53	97.85 ± 7.83
Blood Urea (mg/dl)	20.5 ± 4.72	25.1 ± 6.84	22.6 ± 6.58	19.31 ± 5.83	20.31 ± 6.83	19.81 ± 8.72
Serum Creatinine (mg/dl)	0.41 ± 0.15	0.42 ± 0.15	0.45 ± 0.14	0.43 ± 0.15	0.45 ± 0.15	0.42 ± 0.14
Sodium (mEq/L)	137 ± 7.58	139.8 ± 3.04	136.3 ± 4.83	132.6 ± 5.25	133.6 ± 5.31	132.5 ± 3.78
Potassium (mEq/L)	4.64 ± 0.65	4.26 ± 0.33	4.4 ± 0.21	4.93 ± 0.45	4.87 ± 0.93	4.75 ± 1.43

Abbreviations: *n*: number of samples, ALT: Serum glutamic oxaloacetic transaminase, SGPT: Serum glutamic pyruvic transaminase.

## Data Availability

The data in this study can be availed by request from the corresponding author.

## Supplementary Materials

The following supporting information can be downloaded at: https://www.mdpi.com/article/10.3390/vaccines11010100/s1. S1: Physico-chemical characterization of the as-synthesized AmB-NA. S2: Reverse Transcription Polymerase Chain Reaction (RT-PCR) for determination of cytokine concentration. S3: Determination of nitrite and level of iNOS expression. S4: Assessment of reactive oxygen species (ROS) through ELISA.

## Abbreviations

ALT: Alanine transaminase; AmB: Amphotericin B; AmB-NA: Amphotericin B nanocrystal assemblies; AST: Aspartate transaminase; BUN: Blood urea nitrogen; DLS: Dynamic light scattering; DMSO: Dimethyl sulfoxide; DTH: Delayed type of hypersensitivity; MTT: (3-(4,5-dimethylthiazol-2-yl)-2,5-diphenyltetrazolium bromide; PEC: Peritoneal exudate cell; RBC: Red blood cell; RT-PCR: Reverse transcriptase-polymerase chain reaction; SLA: Soluble leishmanial antigen; TEM: Transmission electron microscopy; VL: Visceral leishmaniasis.
