# Supplementary material for: Amphotericin B Nano-Assemblies Circumvent Intrinsic Toxicity and Ensure Superior Protection in Experimental Visceral Leishmaniasis with Feeble Toxic Manifestation
Source: Vaccines (Basel). 2023 Jan 1;11(1):100. doi: 10.3390/vaccines11010100 (PMC9866558; doi:10.3390/vaccines11010100)
Supplement: Supplementary file 1 [file vaccines-11-00100-s001.zip › vaccines-2061492-supplementary.pdf]

Amphotericin B nano-assemblies circumvent intrinsic toxicity and ensure superior protection in experimental visceral leishmaniasis with feeble toxic manifestation

Supplementary Materials

Physico-chemical characterization of the as-synthesized AmB-NA

The particle size of as-synthesized AmB-NA was measured by photon correlation spectroscopy (90Plus/BI-MAS, Brookhaven Instruments, Holtville, NY, USA) as described previously [26]. For the measurement of size, samples were diluted in Milli-Q water and monitored for at least 120 seconds. Nanoparticles were used at a concentration of 10^{-4} M. All measurements were carried out in triplicate.

Furthermore, measurement of dynamic light dispersion was conducted using DynaPro-TC-04 equipment (Protein Solutions, Wyatt Technology, Santa Barbara, CA, USA) installed with a temperature-controlled micro sampler. Briefly, the as-synthesized AmB-NA nanoparticles-based formulations (2 mg/mL) were filtered directly into a 12 µL quartz cuvette using 0.22 µm and 0.02 µm Whatman syringe filters. Twenty measurements were taken for each test. Mean hydrodynamic radius (R_h) was calculated at optimal resolution using Dynamics technology [26]. Based on an autocorrelation study of scattered light intensity information, the R_h was estimated based on a Stokes-Einstein relationship translation diffusion coefficient:

$$R_h = \frac{kT}{6\pi\eta D}$$

where R_h is the hydrodynamic radius, k is the Boltzmann constant, T is temperature, η is the viscosity of water, and D is the diffusion coefficient.

DTS software was used to determine the zeta potential of nanoparticles (Malvern Instruments Limited, Malvern, UK) based on M3-PALS technology. The sample was filtered using a 0.22 μm polyethersulfone syringe filter (Millipore, Bangalore, India). The formulation was lyophilized, followed by reconstitution in a 20 mM phosphate buffer (pH 7.4). The average zeta potential was calculated thrice.

The morphology and size (TEM analysis) of the as-synthesized AmB-NA nanoparticles was characterized using an electron microscope (CM-10, Philips, Hamburg, Germany). AmB-NA nanoparticles (10 μg/mL in MilliQ water) were lyophilized. The lyophilized AmB-NA nanoparticles were suspended in 20 mM phosphate-buffered saline (PBS, pH 7). A single drop of AmB-NA suspension was mounted on a clear glass stub, air-dried, and coated with gold-palladium by means of sputter coater. An accelerating voltage of 20 kV was used for TEM imaging.

The as-synthesized AmB-NA was scanned spectro-photometrically (300–450 nm) on a double beam spectrophotometer (model V-750, Jasco Inc, Easton, MD, USA) operated at a resolution of 1 nm. The analysis of both Fungizone and the AmB−NA was executed employing the same concentration of both formulations (10^{-5} M). We also performed FTIR spectroscopic studies of as-synthesized AmB nanoparticles employing a Perkin-Elmer FTIR Spectrum One spectrophotometer. FTIR Spectrum One spectrophotometer in the diffuse reflectance mode operating at a resolution of 4 cm^{-1}. Each sample was scanned three times to check the authenticity of the data. The spectra were taken between 4000 cm^{-1} and 1000 cm^{-1} by averaging 128 scans for each spectrum.

Reverse Transcription Polymerase Chain Reaction (RT-PCR) for determination of cytokine concentration

Furthermore, variation in the expression level of various cytokines was further validated by a semi-quantitative reverse transcription-polymerase chain reaction (RT-PCR). For this, forward and reverse primers for IFN-γ, TNF-α, and IL-10 were synthesized (IDT, India) following the sequence mentioned elsewhere [44]. Likewise, the primer sequence for the TNF-α cytokines was taken from the OriGene website (Rockville, MD, USA). According to the manufacturer's instruction, reverse transcription was executed using 1μg of total RNA employing a cDNA synthesis kit (Roche, USA). The synthesized cDNA was amplified by PCR for specified genes such as IL-12, IL-10, IFN-γ, TNF-α, and GAPDH, etc.[44] The primer for IFN-γ, IL-10, TNF-$\propto$, and GAPDH were as follows: Forward $_{IFN\gamma}$: ATGAACGCTACACACTGCAT, Reverse $_{IFN\gamma}$:

AGTCTGAGGTAGAAAGAGAT, Forward $_{IL-10}$: GGTGCCTATGTCTCAGCCTCTT Reverse $_{IL-10}$: CCATAGAACTGATGAGAGGGAG, Forward $_{TNF-\alpha}$: ATGCCTGGCTCAGCACTGCT Reverse $_{TNF-\alpha}$: TAACCCTTAAAGTCCTGCAT, Forward $_{GAPDH}$: TGCATCCTGCACCACCAACT, and Reverse $_{GAPDH}$: TGGGATGACCTTGCCCACAG, respectively. GAPDH was used as a loading control. The PCR mixture (25 μl) contains 0.5μM of forward and reverse primer, 0.5mM of each dNTP, 2 mM MgCl2, 0.5 μg of synthesized cDNA, and 1 μl polymerase. Details regarding the sequence of PCR primers, annealing temperature, and PCR product size were done with few modifications performed elsewhere [44]. The PCR was performed for 28 cycles where parameters for each cycle are as follows: (a) denaturation at 95°C for 30 sec, (b) annealing (ranging from 55-62°C) for 30 sec, and (c) extension at 72°C for 45 sec. The sample was preheated at 95 °C for 3 min before PCR. The PCR product was run on 1.5% agarose gel, stained with ethidium bromide (0.5 μg/ml). The associated Gene-tool software (Syngene, USA) and gel documentation system were utilized to quantify PCR product.

Determination of nitrite and level of iNOS expression

To explicate the consequence of bioactive fraction on nitric oxide release, freshly isolated mononuclear splenocytes cells were plated in 12 well microtiter culture plate (1×10^6 cells/mL/well. Cells were stimulated with SLA (20 μg/mL) and incubated in the CO_2 incubator at 37 ºC for 48 h. Following incubation, cell-free culture supernatants were isolated and stored at -70 ºC for further use. The level of nitric oxide (NO) induced in the cultivated cells was quantified using a nitric oxide assay kit (Thermo Fisher Scientific, EMSNO). The NO levels were deduced by using a standard sodium nitrite concentration curve. The minimum detectable concentration of nitrite employing the kit was 0.222 mM. Coherently, iNOS concentration was determined by semi-quantitative RT-PCR[48] using primers Forward $_{iNOS}$: AGGAGGAGAGAGATCCGATTTAG Reverse $_{iNOS}$: TCAGACTTCCCTGTCTCAGTAG Forward $_{GAPDH}$: TGCATCCTGCACCACCAACT, and Reverse $_{GAPDH}$: TGGGATGACCTTGCCCACAG, respectively.

Assessment of reactive oxygen species (ROS) through ELISA

Reactive oxygen species (ROS) were assessed by ELISA utilizing a cell permeant 0.4mM H_2DCFDA (2',7'-dichlorodihydrofluorescein diacetate) and lysis buffer. Cultured cell was suspended in 500 μl PBS. 0.4mM H_2DCFDA was added followed by 15 min incubation. Cells were pellet down and washed with 1X PBS. Thereafter, cells were lysed using 300 μl Lysis buffer. Following centrifugation supernatant was transferred to 96 well plate. The concentration of ROS was quantified using ELISA reader at 504 nm.
